# Update on the diagnosis and treatment of neuromyelitis optica spectrum disorders (NMOSD) – revised recommendations of the Neuromyelitis Optica Study Group (NEMOS). Part II: Attack therapy and long-term management

**DOI:** 10.1007/s00415-023-11910-z

**Published:** 2023-09-07

**Authors:** Tania Kümpfel, Katrin Giglhuber, Orhan Aktas, Ilya Ayzenberg, Judith Bellmann-Strobl, Vivien Häußler, Joachim Havla, Kerstin Hellwig, Martin W. Hümmert, Sven Jarius, Ingo Kleiter, Luisa Klotz, Markus Krumbholz, Friedemann Paul, Marius Ringelstein, Klemens Ruprecht, Makbule Senel, Jan-Patrick Stellmann, Florian Then Bergh, Corinna Trebst, Hayrettin Tumani, Clemens Warnke, Brigitte Wildemann, Achim Berthele, The Neuromyelitis Optica Study Group (NEMOS), Philipp Albrecht, Klemens Angstwurm, Susanna Asseyer, Ana Beatriz Ayroza Galvao Ribeiro Gomes, Antonios Bayas, Stefanie Behnke, Stefan Bittner, Franziska Buetow, Mathias Buttmann, Ankelien Duchow, Daniel Engels, Thorleif Etgen, Katinka Fischer, Benedikt Frank, Anna Gahlen, Achim Gass, Johannes Gehring, Christian Geis, Ralf Gold, Yasemin Göreci, Jonas Graf, Sergiu Groppa, Matthias Grothe, Julia Gutbrod, Kersten Guthke, Axel Haarmann, Maria Hastermann, Bernhard Hemmer, Mariella Herfurth, Marina Herwerth, Frank Hoffmann, Olaf Hoffmann, Martin W Hümmert, Leila Husseini, Jutta Junghans, Matthias Kaste, Peter Kern, Karsten Kern, Pawel Kermer, Christoph Kleinschnitz, Wolfgang Köhler, Kimberly Körbel, Markus Kowarik, Markus Kraemer, Julian Kretschmer, Natalia Kurka, Theodoros Ladopoulus, Ann-Sophie Lauenstein, Sarah Laurent, De-Hyung Lee, Dominik Lehrieder, Frank Leypoldt, Martin Liebetrau, Ralf Linker, Gero Lindenblatt, Lisa Lohmann, Felix Lüssi, Peter Luedemann, Michelle Maiworm, Martin Marziniak, Christoph Mayer, Stefanie Meister, Mathias Mering, Imke Metz, Sven Meuth, Jasmin Naumann, Oliver Neuhaus, Tradite Neziraj, Moritz Niederschweiberer, Sabine Niehaus, Carolin Otto, Florence Pache, Thivya Pakeerathan, Sarah Passoke, Marc Pawlitzki, Hannah Pellkofer, Mosche Pompsch, Anne-Katrin Pröbstel, Refik Pul, Sebastian Rauer, Nele Retzlaff, Arne Riedlinger, Paulus Rommer, Veith Rothhammer, Kevin Rostásy, Rebekka Rust, Christoph Ruschil, Matthias Schwab, Maria Seipelt, Patrick Schindler, Carolin Schwake, Patricia Schwarz, Claudia Sommer, Alexander Stefanou, Till Sprenger, Andreas Steinbrecher, Heike Stephanik, Muriel Stoppe, Klarissa Stürner, Marie Süße, Athanasios Tarampanis, Simone Tauber, Daria Tkachenko, Annette Walter, Klaus-Peter Wandinger, Anna Walz, Martin Weber, Jens Weise, Jonathan Wickel, Heinz Wiendl, Alexander Winkelmann, Yavor Yalachkov, Uwe Zettl, Ulf Ziemann, Frauke Zipp

**Affiliations:** 1 Institute of Clinical Neuroimmunology, LMU Hospital, Ludwig-Maximilians-Universität München, Munich, Germany; 2 Department of Neurology, School of Medicine, Technical University Munich, Klinikum Rechts der Isar, Munich, Germany; 3 Department of Neurology, Medical Faculty, Heinrich Heine University Düsseldorf, Düsseldorf, Germany; 4 Department of Neurology, St. Josef Hospital, Ruhr University Bochum, Bochum, Germany; 5 Department of Neurology, Charité—Universitätsmedizin Berlin, corporate member of Freie Universität Berlin and Humboldt-Universität zu Berlin, Berlin, Germany; 6 Experimental and Clinical Research Center, a cooperation between the Max Delbrück Center for Molecular Medicine in the Helmholtz Association and Charité—Universitätsmedizin Berlin, Berlin, Germany; 7 Max Delbrück Center for Molecular Medicine in the Helmholtz Association (MDC), Berlin, Germany; 8 NeuroCure Clinical Research Center, Charité Universitätsmedizin Berlin, corporate member of Freie Universität Berlin and Humboldt-Universität Zu Berlin, and Berlin Institute of Health, and Max Delbrück Center for Molecular Medicine, Berlin, Germany; 9 Department of Neurology and Institute of Neuroimmunology and MS (INIMS), University Medical Center Hamburg-Eppendorf, Hamburg, Germany; 10 Department of Neurology, Hannover Medical School, Hannover, Germany; 11 Molecular Neuroimmunology Group, Department of Neurology, University of Heidelberg, Heidelberg, Germany; 12 Marianne-Strauß-Klinik, Behandlungszentrum Kempfenhausen für Multiple Sklerose Kranke, Berg, Germany; 13 Department of Neurology with Institute of Translational Neurology, University of Münster, Münster, Germany; 14 Department of Neurology and Pain Treatment, Immanuel Klinik Rüdersdorf, University Hospital of the Brandenburg Medical School Theodor Fontane, Rüdersdorf bei Berlin, Germany; 15 Faculty of Health Sciences Brandenburg, Brandenburg Medical School Theodor Fontane, Rüdersdorf bei Berlin, Germany; 16 Department of Neurology & Stroke, University Hospital of Tübingen, Tübingen, Germany; 17 Department of Neurology, Center for Neurology and Neuropsychiatry, LVR-Klinikum, Heinrich Heine University Düsseldorf, Düsseldorf, Germany; 18 Department of Neurology, University of Ulm, Ulm, Germany; 19 APHM, Hopital de la Timone, CEMEREM, Marseille, France; 20 Aix Marseille University, CNRS, CRMBM, Marseille, France; 21 Department of Neurology, University of Leipzig, Leipzig, Germany; 22 Department of Neurology, Faculty of Medicine, University Hospital Cologne, University of Cologne, Cologne, Germany

**Keywords:** Neuromyelitis optica spectrum disorders (NMOSD), Attacks, Disability, Aquaporin-4 (AQP4), Double-negative NMOSD, Immunotherapies, Long-term management

## Abstract

This manuscript presents practical recommendations for managing acute attacks and implementing preventive immunotherapies for neuromyelitis optica spectrum disorders (NMOSD), a rare autoimmune disease that causes severe inflammation in the central nervous system (CNS), primarily affecting the optic nerves, spinal cord, and brainstem. The pillars of NMOSD therapy are attack treatment and attack prevention to minimize the accrual of neurological disability. Aquaporin-4 immunoglobulin G antibodies (AQP4-IgG) are a diagnostic marker of the disease and play a significant role in its pathogenicity. Recent advances in understanding NMOSD have led to the development of new therapies and the completion of randomized controlled trials. Four preventive immunotherapies have now been approved for AQP4-IgG-positive NMOSD in many regions of the world: eculizumab, ravulizumab - most recently-, inebilizumab, and satralizumab. These new drugs may potentially substitute rituximab and classical immunosuppressive therapies, which were as yet the mainstay of treatment for both, AQP4-IgG-positive and -negative NMOSD. Here, the Neuromyelitis Optica Study Group (NEMOS) provides an overview of the current state of knowledge on NMOSD treatments and offers statements and practical recommendations on the therapy management and use of all available immunotherapies for this disease. Unmet needs and AQP4-IgG-negative NMOSD are also discussed. The recommendations were developed using a Delphi-based consensus method among the core author group and at expert discussions at NEMOS meetings.

**Supplementary Information:**

The online version contains supplementary material available at 10.1007/s00415-023-11910-z.

## Introduction

Neuromyelitis optica spectrum disorder (NMOSD) is a rare and severe inflammatory autoimmune disease of the central nervous system (CNS) that was identified as a distinct clinical entity with the discovery of aquaporin-4 immunoglobulin G antibodies (AQP4-IgG) [90, 124, 137]. The 2015 international consensus-based diagnostic criteria are currently used to diagnose NMOSD. Applying these criteria, AQP4-IgG-positive NMOSD appears relatively straightforward to diagnose, but identifying AQP4-IgG-negative patients with these criteria is also possible [245].

Without proper treatment, patients with NMOSD may develop significant disability over time due to the recurrence of attacks and insufficient recovery from severe disease attacks [92, 108, 111]. Currently, there is no known curative treatment for NMOSD; therefore, the main goals of therapy are to counteract acute attacks promptly and effectively and to prevent future attacks by initiating immunotherapy as soon as a definite diagnosis of NMOSD is established. Recently, several prospective randomized controlled trials (RCT) have led to FDA approval of the first three immunotherapies for patients with AQP4-IgG-positive NMOSD: eculizumab in June 2019, inebilizumab in June 2020, and satralizumab in August 2020 [51, 179, 223, 250]. In addition, rituximab was approved for NMOSD in Japan in June 2022 based on the results of an investigator-initiated phase II/III clinical study [216], and in May 2023, the EMA approved ravulizumab for the treatment of AQP4-IgG-positive NMOSD.

The Neuromyelitis Optica Study Group (NEMOS), founded in 2008 as a nationwide network of primary, secondary, and tertiary care centers in Germany, aims to improve the care of patients with NMOSD (www.nemos-net.de) [92, 225], and the group updated its recommendations on the diagnosis and differential diagnosis of NMOSD in 2023 [88]. Interestingly, an international panel of clinical experts has very recently published a Delphi consensus on the management of AQP4-IgG-positive NMOSD, which focuses on recommendations for eculizumab, inebilizumab, and satralizumab [176]. In this article, the NEMOS group now provides its updated practical recommendations for the treatment of NMOSD, which cover the management of acute attacks and preventing future attacks through immunotherapy, including approved and established off-label treatments. These recommendations are based on current literature and the expert opinions of clinical care providers specialized in NMOSD, who are all members of NEMOS. Myelin–oligodendrocyte–glycoprotein (MOG)-IgG-associated disease (MOGAD) is now considered a distinct disease entity; therefore, recommendations for therapy in patients with MOGAD will be addressed separately [20, 89]. Patients with NMOSD who are negative for both AQP4-IgG and MOG-IgG in serum are described as “double-negative” throughout the manuscript.

## Methods

The initial version of this manuscript was prepared by TK, KG, and AB as part of a core working group of 24 German neurologists from 15 NEMOS centers, all specialized in the management of NMOSD, through discussions at NEMOS meetings and sessions. The manuscript was then reviewed and edited by representatives of the group, and specific recommendations were developed by the core group through the Delphi method [159]. The revised version of the manuscript, including the recommendations, was distributed to all members of NEMOS (see Appendix) for further feedback and revisions. Survey results are presented in the supplementary material.

## AQP4-IgG-positive NMOSD: from pathophysiology to therapy

Autoantibodies against AQP4 are present in more than 80% of NMOSD patients and play a central role in disease pathogenesis [94]. An increased understanding of the pathophysiology of AQP4-IgG-positive NMOSD has led to the development of targeted therapeutic approaches. Lesions in AQP4-IgG-positive NMOSD patients are characterized by mixed immune cell infiltrates as well as IgG and complement (C9neo) deposition around blood vessels, associated with macrophage, neutrophil, and eosinophil infiltration [137, 138]. Complement-mediated enhancement of local inflammation and cytotoxicity are key elements in the disease process [125, 127, 128, 173]. These inflammatory reactions lead to loss of AQP4 expression on astrocytes, particularly at the blood–brain barrier, resulting in astrocytic loss and secondary damage to oligodendrocytes and neurons.

In addition to nonspecific immunosuppressants, such as oral glucocorticoids, azathioprine or mycophenolate mofetil, more tailored therapeutic approaches have been developed that specifically target the pathophysiology of the disease. Depletion of B cells through anti-CD20 antibodies such as rituximab has been successfully used as a treatment of NMOSD for more than 15 years [50], confirming the central role of B cells in the disease [48, 54, 244]. Recently, the monoclonal anti-CD19 antibody inebilizumab, which, in addition to B cells, also targets CD19-expressing antibody-producing plasmablasts, has been shown to be beneficial in AQP4-IgG-positive NMOSD [33, 209]. In addition, inhibiting the proinflammatory interleukin-6 (IL-6) pathway through drugs such as tocilizumab and satralizumab has also been effective in treating NMOSD by addressing blood–brain barrier dysfunction, CNS infiltration of immune cells, B cell differentiation into plasmablasts and plasma cells with increased AQP4-IgG secretion, and B cell survival [41, 70, 195, 219]. Moreover, inhibition of the terminal complement cascade by the C5 inhibitors eculizumab and ravulizumab has been effectively used for attack prevention, highlighting the central role of autoantibody-mediated complement activation in the final steps of NMOSD pathophysiology [14].

## Treatment of NMOSD: general aspects and outcome measures

Outcome measures and attack definitions used in most studies of NMOSD have been developed for multiple sclerosis (MS) and have not been validated for NMOSD [240]. Since several features differentiate MS from NMOSD, such as the absence of progressive disease independent of acute attacks, more severe attacks, and mostly no informative changes on magnetic resonance imaging (MRI) over time, outcome measures used for MS are challenging to directly apply to NMOSD. As there are no evidence-based definitions of neuromyelitis optica attacks, elaborate definitions have been proposed in the context of placebo-controlled studies; however, they are yet to be standardized [49]. In addition, there is no validated method to evaluate attack severity. The expanded disability score (EDSS) and functional system scores (FSS) were adopted from MS to measure disability in NMOSD. Nevertheless, some dimensions, such as visual function, pain, fatigue, depression, cognition, and function of upper limbs are not adequately represented within the EDSS. Low contrast visual acuity as well as specific visual function questionnaires, such as the National Eye Institute-Visual Function Questionnaire, might be additional helpful tools to detect and monitor visual impairment [175]. Moreover, there is a lack of randomized controlled trials on the treatment of NMOSD attacks. While current definitions of NMOSD attacks may vary, they generally involve the emergence of new neurological symptoms and/or exacerbation of pre-existing ones that persist for more than 24 h, with or without new/enlarging or enhancing MRI lesions. Pseudo-attack, on the other hand, refers to a worsening of pre-existing neurological symptoms that can be attributed to other clinical factors, such as infections, fever, injury, comorbidities, adverse reactions to medications, change in mood, pain, or dysautonomia [100].

### How to treat attacks?

Without treatment, the outcome of NMOSD attacks is often poor, with full recovery observed in only a minority of patients [92, 111]. However, recovery can be improved by prompt initiation of therapy in acute attacks and early escalation [5, 111]. Factors such as pre-existing disability, type and severity of an attack, age, and, most importantly, time interval to treatment initiation also contribute to recovery [19, 84, 110, 111, 131, 214].

The standard of care for acute attacks in both AQP4-IgG-positive and double-negative NMOSD are high-dose glucocorticoids and apheresis therapy. Methylprednisolone (MP) should be usually administered intravenously (i. v.) at a dose of 1000 mg per day for 3–5 days, followed by an oral MP taper (starting with either 1 mg/kg/day or 20–30 mg/day and then tapered to 10–15 mg/day within 2–3 weeks) in combination with proton pump inhibition and thrombosis prophylaxis. Low-dose oral glucocorticoids for up to 3–6 months are also considered beneficial for preventing subsequent early attacks, although there is a lack of prospective studies [232]. The duration of low-dose add-on glucocorticoid treatment depends on the AQP4-IgG serostatus, disease activity, mode of action, and the expected time to the attack-preventive effect of the subsequent immunotherapy.

If, within the first days, patients do not respond sufficiently to MP, rescue treatment with apheresis therapy, such as therapeutic plasma exchange (PE) or immunoadsorption (IA), should be administered early on [30, 111, 210, 241]. Most studies performed an average of 5 cycles daily or every other day, but up to 10 cycles may be applied [170]. IA has been used less frequently, and no clear difference has been established between PE and IA in terms of therapeutic outcomes, but data are limited, and more experience exists for PE [28, 110].

**Fig. 1:**
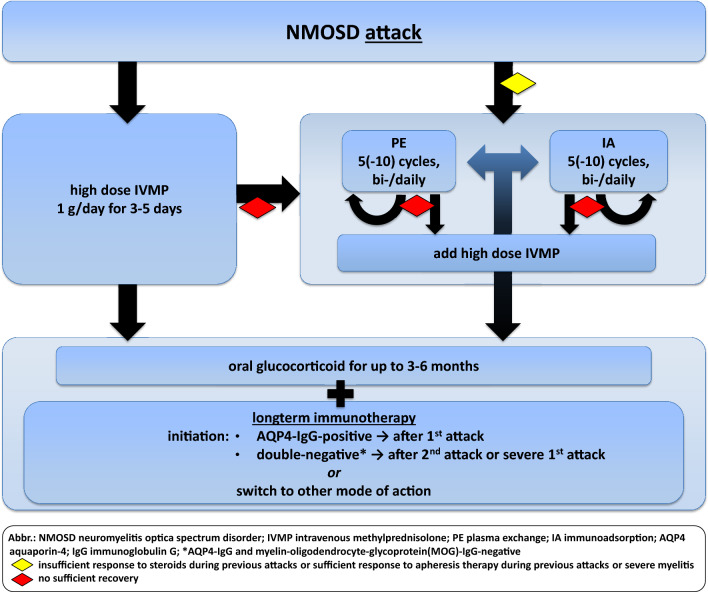
Attack therapy

Several retrospective studies have shown that an early start of apheresis therapy is associated with better outcomes in NMOSD patients [29, 56, 110]. When apheresis therapy was started within 0–2 days of symptom onset without delay, up to 40% of patients showed complete remission; however, with a later treatment starting ≥ 7 days after symptom onset, only 3.7% of patients completely recovered [29, 56, 110]. Therefore, early adjunctive (add-on to glucocorticoids) apheresis therapy should be considered in patients with severe attacks. Using apheresis as the only first-line treatment without high-dose glucocorticoids was advantageous for patients with the presence of myelitis in one study [111]. A repeated treatment course has also been shown to improve outcomes and lower the number of non-responders [111]. Thus, patients may benefit from repeated therapy courses (apheresis and/or glucocorticoids) if they insufficiently responded or continued exhibiting functional deficits after the first or second treatment course for NMOSD attacks (Fig. 1). Other experimental therapy approaches, such as intravenous immunoglobulins (IVIG), early anti-CD20 therapy, and early anti-complement therapy, have been reported in single case series to possibly lead to favorable outcomes in acute attacks [65, 127, 132, 147]. Future therapies may also include antibodies against the neonatal Fc receptor, which are currently being studied in an open-label trial [236, ClinicalTrials.gov].

**Box 1: Recommendation—attack therapy** **A1** Attack therapy must be initiated as early as possible in NMOSD attacks.

**A2** Following attack therapy (high-dose glucocorticoid therapy and/or apheresis therapy), an oral taper of glucocorticoids should be administered for up to 3–6 months* to prevent subsequent attacks, especially at the start or switch of long-term immunotherapy.

**A3** Patients who do not show sufficient recovery after high-dose glucocorticoids should begin apheresis therapy early.

**A4** Apheresis therapy may be the first-line treatment option for patients with:

- insufficient response to glucocorticoids during previous attacks
- sufficient response to apheresis therapy during previous attacks
- severe myelitis.

**A5** Either plasma exchange or immunoadsorption may be used as apheresis therapy.

**A6** For patients with severe attacks, concomitant treatment with high-dose glucocorticoids and apheresis may be used.

*duration of low-dose add-on glucocorticoid treatment depends on the AQP4-IgG serostatus, disease activity, mode of action and the expected time to the attack-preventive effect of the subsequent immunotherapy.

### How to prevent attacks?

The second main goal of NMOSD therapy is to prevent further attacks. Unlike MS, disability in NMOSD mainly results from poor and incomplete recovery from clinical attacks [56, 92, 111, 154, 247]. AQP4-IgG-positive NMOSD typically takes a relapsing course in almost all cases, with patients having a particularly high risk for attacks in the year following onset or any subsequent attack. However, some patients may experience several years and even decades without attacks [4, 242]. Risk factors for attacks and disability in AQP4-IgG-positive NMOSD include the age of onset, sex, ethnicity, onset attack phenotype, and treatment [171]. Studies have shown that the risk of attacks is similar between AQP4-IgG-positive and AQP4-IgG-negative patients, even when comparing strictly double-negative and AQP4-IgG-positive NMOSD [96, 206]. As every attack can leave a patient permanently disabled and worsen health-related quality of life, early initiation of immunotherapy is crucial to prevent further clinical episodes and avoid long-term neurological sequelae [25, 148].

Before 2019, recommendations on the long-term treatment of NMOSD, including ours, were mainly based on retrospective studies, case series, open-label trials, and expert opinions without any approved immunotherapies. The serostatus (AQP4-IgG-positive vs. double-negative) was not considered in treatment algorithms. Azathioprine, mycophenolate mofetil, or rituximab and in some countries also oral glucocorticoids were commonly used as first-line agents for both AQP4-IgG-positive and -negative patients, with the reduction of attack rates analyzed irrespective of the serostatus in retrospective studies. In multiple non-RCTs, rituximab was shown to be superior to mycophenolate mofetil and azathioprine, and it has become the preferred first-choice therapy in recent years [83, 149, 160]. The IL-6 receptor (IL-6-R) antibody tocilizumab has also been increasingly used as rescue therapy, showing benefits in several case series [12, 194, 195].

Four therapies, eculizumab, inebilizumab, and satralizumab and most recently ravulizumab have been approved for use in AQP4-IgG-positive NMOSD since 2019. Rituximab was also approved for NMOSD in Japan in 2022 following the positive results of the placebo-controlled RIN-1 trial [216]. Nevertheless, inclusion criteria and methods varied considerably between studies and indications for use do not fully reflect study populations. The order of preference for these therapies is yet unclear, and further comparative trials and real-world data are needed. In addition, outside of clinical studies, fatalities have been reported during treatment with these new therapies [193]. However, clinical data on these cases are not yet published, and causality and mortality risks are still unknown.

In the following section, we summarize and discuss specific therapies that have been widely used or recently approved and provide specific recommendations for choosing, initiating, and maintaining therapy regimes. For an overview, see also Table 1.

**Table 1:** Recommendations on the application, monitoring, and important risks of preventive immunotherapies in NMOSD^1,2^

Drug	Application	Full onset of action	Common side effects	Risk of infections	Other important risks	Blood monitoring	Additional suggested monitoring
Azathioprine	Oral, daily, 2.5–3.0 mg/kg body weight	After 6–12 months	Hematological abnormalities (lymphocytopenia, pancytopenia), elevation of liver enzymes, gastrointestinal side effects	Upper respiratory tract and urinary tract infections, opportunistic infections	Drug-induced fever, several drug interactions (including allopurinol, anti-viral and anti-coagulatory drugs), photosensitization (skin), increased cancer risk with duration of treatment (> 10 years)	BCC and differential WBCC, liver enzymes	Regular screening for cancer (by dermatologist, gynecologist)
Mycophenolate mofetil	Oral, daily, 1000–2000 mg	After 8–12 weeks	Hematological abnormalities (anemia), elevation in liver enzymes, gastrointestinal side effects	Upper respiratory tract and urinary tract infections, opportunistic infections	Increased cancer risk, teratogenic and embryotoxic effects	BCC and differential WBCC, liver enzymes	Regular screening for cancer (by dermatologist, gynecologist)
Glucocorticoids	For attack: i. v., 1x/day, 1000–2000 mg, over 3–5 days For taper: oral, 1x/day, starting with 1 mg/kg/day or 20–30 mg/day and then tapered to 10–15 mg within 2–3 weeks; For long-term use: oral, 1x/day, individual dosing (ideally ≤ 7.5 mg/day)	Immediate effects	Hematological abnormalities (lymphopenia), elevation in liver enzymes	Increased risk for infections, including opportunistic infections (e.g., PJP, particularly if administered with other IS)	Diabetes, arterial hypertension, osteoporosis, cataract, adrenal insufficiency, Cushing’s syndrome	BCC and differential WBCC, liver enzymes, electrolytes, blood glucose	Use of glucocorticoids in combination with proton pump inhibition and thrombosis prophylaxis Monitoring of blood pressure; with long-term use: bone densitometry, eye examination, cardiovascular check-ups
Rituximab	i. v., with premedication (glucocorticoids, antiphlogistics, antihistamines); usually initially 1000 mg at day 1 and day 14, followed by 500–1000 mg every 6 months	B-cell depletion within 4 weeks, full onset of action after 8–12 weeks	Nausea, exanthema, headache	Upper respiratory tract and urinary tract infections, hepatitis B reactivation, opportunistic infections (including PML), no PML in NMOSD reported so far	Infusion-related pseudo-allergic reactions (due to cell lysis), leucopenia, neutropenia and LON, hypogammaglobulinemia	Differential WBCC, serum immunoglobulins, CD19/20-positive B-cell count	Monitoring for infusion-related (allergic) reactions
Inebilizumab	i. v., with premedication (glucocorticoids, antiphlogistics, antihistamines); initially 300 mg at day 1 and day 14, followed by 300 mg every 6 months	B-cell depletion within 2 weeks, full onset of action after 6–8 weeks	Arthralgias, back pain	Upper respiratory tract and urinary tract infections, opportunistic infections (including PML)	Infusion-related pseudo-allergic reactions (due to cell lysis), lymphopenia, neutropenia and LON, hypogammaglobulinemia	Differential WBCC, serum immunoglobulins, CD19/20-positive B-cells count	Monitoring for infusion-related (allergic) reactions
Eculizumab	i. v., 900 mg 1x/week weeks 0–3, 1200 mg 1x/week week 4, thereafter 1200 mg every 2 weeks	Immediate within 1–2 weeks	Headaches, upper respiratory tract infections	Meningococcal infection and infections with other encapsulated bacteria	Anemia, leukopenia, fungal infections, infusion-related (allergic) reactions	BCC and differential WBCC	Patient teaching and close monitoring for meningococcal infection (exclusion before each infusion)
Ravulizumab	i. v., weight-based^2^, loading dose of 2400–3000 mg on days 1 and 15 followed by 3000–3600 mg once every 8 weeks	Immediate within 1–2 weeks	Headaches, upper respiratory tract infections	Meningococcal infection and infections with other encapsulated bacteria	Anemia, leukopenia, fungal infections, infusion-related (allergic) reactions	BCC and differential WBCC	Patient teaching and close monitoring for meningococcal infection (exclusion before each infusion)
Tocilizumab	i. v., 6–8 mg/kg body weight, every 4–6 weeks*	After 12 to 24 weeks	Injection-related reactions, headache	Upper respiratory tract and urinary tract infections	Neutropenia, thrombocytopenia, elevation in liver enzymes, flare-up of chronic diverticulitis with potential gastrointestinal perforations, elevations in cholesterol or triglycerides, infusion-related (allergic) reactions	BCC and differential WBCC, liver enzymes, lipids	Clinical monitoring for infections due to suppression of CRP production in the context of infections
Satralizumab	s. c., 120 mg at weeks 0, 2, and 4, followed by every 4 weeks	After 12 to 24 weeks	Injection-related reactions, headache, arthralgia,	Mild to moderate infections, no opportunistic infections so far reported	Neutropenia, thrombocytopenia, elevation in liver enzymes, elevations in cholesterol or triglycerides, decrease in C3, C4 and fibrinogen	BCC and differential WBCC, liver enzymes, lipids	Clinical monitoring for infections due to suppression of CRP production in the context of infections

*BCC* blood cell count, *CRP* C-reactive protein, *IS* immunosuppressive therapies, *i. v.* intravenously, *LON* late-onset neutropenia, *PJP* pneumocystis jirovecii pneumonia, *PML* progressive multifocal leukoencephalopathy, *s. c.* subcutaneously, *WBCC* white blood cell count

^1^These aggregated recommendations do not include all potential side effects and do not replace the specific product information for each drug; ^2^for details see product information

*switch to s. c. application possible

**Box 2: Recommendation—long-term therapy: principal recommendations** **B1** Long-term immunotherapy must be offered to patients with AQP4-IgG-positive NMOSD already after the first attack.

#### Specific therapies (see also Tables 1, 2, 3)

**Table 2:** Main data of randomized, double-blind, placebo-controlled, time-to-event trials in NMOSD

Drug	Trial/Randomization	Number of patients / AQP4-IgG serostatus	Inclusion criteria		Concomitant immuno-suppression	Attacks n (%) (HR, 95% CI, and/or p)	previous immunotherapies	Duration of treatment in core study/open-label extension
			Previous disease activity	Age [years]				
Rituximab	RIN-1^1^/1:1	38 / all positive; including 11 AQP4-IgG negative patients who previously tested positive	Any history of optic neuritis or myelitis	16–80	No, but oral glucocorticoids, tapered during initiation period	0 vs. 7 (37%); (p = 0.0058)	0% with rituximab, other n.a	Median 72.1 weeks/mean 20.5 months (SD 10.1)^7^
Inebilizumab	N-MOmentum^2^/3:1	230 / 213-positive, 17-negative	≥ 1 attack in 12 months or ≥ 2 attacks in 24 months	> 18	No, but oral glucocorticoids during initiation period (20 mg/d until d14, then tapered to d21)	21 (12%) vs. 22 (39%);(0.272, 0.15–0.496, *p* < 0.0001)	Inebilizumab group: 66% (mostly azathioprine and glucocorticoids, including 7% with rituximab)	Up to 28 weeks/mean 3.2 years, up to 4.5 years (median)^8^
Eculizumab	PREVENT^3^/2:1	143 / all-positive	≥ 2 attacks in 12 months or ≥ 3 attacks in 24 months with 1 attack in the last 12 months	> 18	Yes	3 (3%) vs. 20 (43%); (0.06, 0.02–0.20, *p* < 0.001)	Eculizumab group: 78% IS at baseline; (27% with previous rituximab)	Median 89.4 weeks/median 132 weeks, up to 277 weeks^9^
Ravulizumab	CHAMPION–NMOSD^4^/Placebo group of PREVENT as external comparator	58 / all-positive	≥ 2 attacks in 12 months or ≥ 3 attacks in 24 months with 1 attack in the last 12 months	> 18	Yes	0 vs. 29 (43%) in PREVENT (0.014, 0.000–0.103, *p* < 0.0001)	Ravulizumab group: 48% IS at baseline; 86% previuos IS (including 36% with previous rituximab)	Median 73.5 weeks (range 11.0–117.7)/OLE ongoing
Satralizumab	SAkuraSky^5^/1:1	83 /55 -positive, 28-negative	≥ 2 attacks in 24 months with 1 attack in the last 12 months	12–74	Yes	8 (20%) vs. 18 (43%); (0.38, 0.16–0.88, *p* = 0.02)	Satralizumab group: 78% with previous IS before add-on IS at baseline (including 4,9% with rituximab);	Median 107.4 weeks/median 4.4 years (range 0.1–7.0)^10^
	SAkuraStar^6^/2:1	95/63-positive, 31-negative	≥ 1 attack in 12 months	18–74	No	19 (30%) vs. 16 (50%); (0.45, 0.23–0.89, *p* = 0.018)	Satralizumab group: 87% with previous IS or other; 13% with previous B-cell depleting therapies	Median 92.3 weeks/median 4.0 years (range 0.1–6.1)^10^

*n.a.* not available, *HR* hazard ratio, *CI* confidence interval, *d* day, *OLE* open-label extension, *SD* standard deviation, *IS* immunosuppressive therapy

^1^Tahara 2020 The Lancet Neurology [216]

^2^Cree 2019 The Lancet [51]

^3^Pittock 2019 N Eng J Med [179]

^4^Pittock 2023 Ann Neurol [178]

^5^Yamamura 2019 N Eng J Med [250]

^6^Traboulsee 2020 The Lancet Neurol [223]

^7^Tahara 2022 MSRD [217]

^8^Rensel 2022 MSJ [188]

^9^ Wingerchuk 2021 Ann Neurol [246]

^10^Yamamura 2022 MSRD [251]

**Table 3:** Summary of the criteria for treatment decision-making in long-term immunotherapy in AQP4-IgG-positive NMOSD

Drug	Application	Full onset of action^1^	Availability^2^	Costs	Additional effects on concomitant rheumatological autoimmune disease	Recommended management/blood monitoring^3^	Effectiveness^4^	Family planning/pregnancy
Azathioprine	Oral, easy and independent	Delayed	+++	Low	Yes	Blood examinations every 1–4 weeks for the first 3 months, followed by three monthly examinations	+	Treatment possible during pregnancy^5^
Mycophenolate Mofetil	Oral, easy and independent	Intermediate	+++	Low	Yes	Blood examinations every 2–4 weeks for the first 3 months, followed by three monthly examinations	+	Teratogenic, must not be used 3 months prior to and during pregnancy
Rituximab	Infusion with premedication and monitoring every 6 months	Intermediate	++	Low (biosimilars), reimbursement issues	Yes	Blood examinations every 4 weeks for the first 3 months, followed by three monthly examinations	+++	Some data for NMOSD available, treatment possible during pregnancy^5^
Inebilizumab	Infusion with premedication and monitoring every 6 months	Intermediate	+	High	n. k	Blood examinations every 4 weeks for the first 3 months, followed by three monthly examinations	+++	No data^5^
Eculizumab	Infusion with monitoring: initially 1x/week, then every 2 weeks; meningococcal vaccination and/or antibiotic prophylaxis before start mandatory**	Rapid	+	Very high	No	Blood examinations after 4 weeks of treatment, followed by three to six monthly examinations; standardized query of meningococcal infection symptoms before each infusion	++++	Limited data, treatment possible during pregnancy after careful risk–benefit evaluation^5^
Ravulizumab	Infusion with monitoring: initially on day 1 and 15, then every 8 weeks; meningococcal vaccination and/or antibiotic prophylaxis before start mandatory**	Rapid	+	Very high	No	Blood examinations after 4 weeks of treatment, followed by three to six monthly examinations; standardized query of meningococcal infection symptoms before each infusion	++++	no data^5^
Tocilizumab	Infusion every 4–6 weeks*	Intermediate	++	Low, reimbursement issues	Yes	Blood examinations every 4 weeks for the first 3 months, followed by three monthly examinations	++	Limited data, treatment possible during pregnancy^5^
Satralizumab	Initial infusion and titration with monitoring, followed by independent self-infusions every 4 weeks	Intermediate	+	High	n. k	Blood examinations every 4 weeks for the first 3 months, followed by three monthly examinations	+++	No data^5^

^1^Full onset of action: delayed: ≥ 6 months, intermediate: 3–6 months, rapid: within 1–3 months

^2^Accessibility: +++ worldwide, ++ in most industrialized countries, + only in specific countries

^3^Please refer to Table 2 for specific laboratory tests to be performed during treatment with the respective substances

^4^Estimates of effectiveness compared to each other, based on the decrease in relapse rates observed in controlled trials (no comparative data), comparative retrospective analyses and meta-analyses: ++++ very high, +++ high, ++ intermediate, + low, n. k. = not known

^5^Considering data availability and limitations, consult the section of the manuscript “Pregnancy and NMOSD”

*Most data are available for intravenous mode of administration, and some data are available for weekly subcutaneous self-administration

**See text for details

##### Classical immunosuppressants: azathioprine, mycophenolate mofetil, low-dose oral glucocorticoids

Oral glucocorticoid therapy broadly suppresses the immune system through both genomic and non-genomic mechanisms by binding to glucocorticoid receptors expressed on lymphocytes. On one hand, low-dose glucocorticoids are used long-term in AQP4-IgG-negative NMOSD patients (often in MOG-IgG-positive patients) as well as in patients with concomitant autoimmune disease, mostly as an adjuvant yet sometimes as monotherapy in countries, where monoclonal antibodies and/or other immunosuppressive therapies are not available. On the other hand, low-dose glucocorticoids are also used for a shorter period as a bridge after an attack until the full effect of subsequent immunotherapy is achieved. There are no controlled data on dosing or the optimal timing for tapering and stopping add-on glucocorticoid therapy. One study reported a safe and effective use of azathioprine combined with low-dose glucocorticoids in Chinese patients with AQP4-IgG-positive NMOSD [185]. Moreover, low-dose glucocorticoid monotherapy (5–15 mg) prevented attacks in some Asian patients with NMOSD in small retrospective studies, showing that attacks are more likely to occur with rapid tapering and doses below 10 mg/day [218, 237]. However, long-term use of glucocorticoids is associated with several side effects, including infections, diabetes mellitus, weight gain, Cushing’s syndrome, and osteoporosis [166] (Table 1). Therefore, a daily prednisolone dose of 7.5 mg or less should ideally be achieved if used as a long-term treatment.

Azathioprine and mycophenolate mofetil have been used as the standard of care in (adult and pediatric) NMOSD for more than 20 years, and they effectively reduce attack rates in both AQP4-IgG-positive and -negative patients, as shown in retrospective studies and non-RCTs [27, 213, 235]. They are widely available and affordable but not approved for NMOSD. Comparisons of effectiveness in the literature are inconsistent, with most studies suggesting that both compounds have comparable efficacy and are similarly inferior to rituximab [39, 74, 160, 183].

Azathioprine, a purine analogue, is used to inhibit the activation and differentiation of lymphocytes. Due to its presumably delayed onset of action, it is often combined with oral glucocorticoids for up to 6 months after initiation of therapy [47]. Thiopurine *S*-methyltransferase polymorphisms appear to modify the risk of side effects [73, 134]. Allopurinol and other xanthine oxidase inhibitors should be avoided as they may increase the plasma levels of active metabolites of azathioprine. Long-term use of azathioprine for neuroimmunological diseases, such as MS or NMOSD, has been studied for more than 20 years, and an increased risk of malignancies is known, such as skin cancer but also lymphoproliferative disorders [45, 122]. One case of progressive multifocal leukoencephalopathy (PML) in a patient with NMOSD under monotherapy with azathioprine has also been reported [67].

Mycophenolate mofetil is a prodrug of mycophenolic acid, which suppresses the proliferation of lymphocytes by inhibiting the inosine-5'-monophosphate dehydrogenase, thereby depleting guanosine nucleotides preferentially in T- and B-lymphocytes. It takes approximately 6–12 weeks for the full therapeutic effect to be seen. It is recommended that mycophenolate mofetil is combined with overlapping oral glucocorticoids for at least the first 3 months of therapy [156]. There is a possibly increased risk of malignancy associated with the use of mycophenolate mofetil, and it must be avoided during pregnancy.

#### B cell depletion

##### Rituximab

Rituximab is a chimeric monoclonal antibody that binds to and depletes CD20-positive B-cells. Its clinical effectiveness in NMOSD has been demonstrated in various retrospective and prospective studies, including AQP4-IgG-positive and -negative (adult and pediatric) patients, with a reduction of attack rates by over 80% [52, 53, 61, 74, 135, 253]. A recent randomized, double-blinded, placebo-controlled trial from Japan (RIN-1) confirmed the efficacy of rituximab in AQP4-IgG-positive NMOSD, although the sample size of the trial was small (Table 2) ([216]. Patients received 375 mg/m^2^ body surface of rituximab i. v. every week for 4 weeks, followed by 6-month interval dosing (1,000 mg every 2 weeks, at 24 weeks and 48 weeks). Based on the results of the trial, rituximab has been recently approved for the treatment of NMOSD in Japan but not in other regions of the world, including Europe and the United States, where it is widely used as an off-label therapy. Therefore, reimbursement by healthcare systems may be an issue in many countries.

The onset of action of B-cell depletion is expected within a few weeks; CD19/20-positive B-cells and CD27 memory cells may be used as surrogate markers for treatment monitoring and re-dosing [1, 22, 105, 217]. As efficient B-cell depletion is essential to avoid attacks and given that most patients exhibit depleted B-cells in the peripheral blood for at least 6 months after a rituximab infusion, re-dosing every 6 months is considered an adequate retreatment frequency. Although the optimal strategy for rituximab re-dosing in NMOSD has yet to be determined, a retreatment dosage of a single infusion at a dose of 500 mg or 1000 mg is often used in a real-world setting [103]. As there is evidence that attacks may occur within the initial 6 months after starting rituximab therapy [22, 37, 76, 207] and the time to full clinical effectiveness is presumed to be delayed, concomitant oral glucocorticoid tapering is recommended after therapy initiation. Anti-drug antibodies (ADA) may affect the effectiveness and should be considered in patients with failing B-cell depletion and ongoing disease activity during the disease course [165]. In addition, fragment c gamma receptor 3A (FCGR3A) polymorphisms (F/F variant) may also impact the response to rituximab [105].

While the use and experience of rituximab in NMOSD comprise more than 15 years, controlled safety data are limited. The safety profile of rituximab is generally acceptable but may depend on factors, such as age, gender, weight, disability, duration of therapy, and history of prior immunosuppressive therapies [22, 107, 228]. Most adverse events observed in the RIN-1 study and its open-label extension (OLE) RIN-2 study were mild and infusion-related (Table 1) [216, 217]. The risk of hypogammaglobulinemia and ensuing infections, which typically comprise infections of the upper respiratory and urinary tract, increases with treatment duration [15, 107, 141, 198, 228]. Serology testing for hepatitis B virus (HBV) is mandatory as there is a potentially life-threatening but avoidable risk of reactivation. Opportunistic infections are rare in rituximab monotherapy, and there are rare cases of PML reported in rituximab-treated cancer patients and patients with rheumatological diseases, such as systemic lupus erythematosus (SLE), as well as in patients receiving additional immunosuppressive therapies, but none in NMOSD [23, 155]. Rare cases of leukopenia and severe neutropenia, or a prolonged B-cell depletion lasting up to several years after rituximab use, have been described in NMOSD patients [182, 192, 205].

**Box 3: Recommendation—long-term therapy: off-label therapies** **B2** For patients who are stable on off-label therapies and have no significant side effects there is no need to be switched to other treatments.

**B3** Conventional immunosuppressive therapies (azathioprine, mycophenolate mofetil, oral glucocorticoids) may be used but are considered less effective than biologicals.

**B4** Low-dose glucocorticoids should not be used as a monotherapy to prevent attacks unless no other options are available.

##### Inebilizumab

Inebilizumab is a humanized monoclonal antibody that binds to and depletes CD19-positive B-cells, including CD19-positive subpopulations of (auto)antibody-producing plasma cells. Data from a phase III randomized placebo-controlled study (*N*-Momentum) and its OLE [51, 142, 188] showed that inebilizumab significantly reduced the attack rate in patients with NMOSD compared to placebo, which led to a preponed end of the blinded phase of the trial [51] (Table 2). Moreover, the trial showed that the risk of an EDSS-based disability progression confirmed after 3 months was lower in patients who received inebilizumab [142]. During the OLE (median 4.5 years), the observed effects of inebilizumab were persistent and even enhanced, with most attacks occurring during the first year of treatment and without new safety concerns [188].

Inebilizumab was initially approved in 2020 in the United States for the treatment of adult patients with AQP4 IgG-positive NMOSD. Approvals have since been granted in many regions of the world, including Europe in 2022 (as monotherapy).The onset of action and clinical effectiveness is unclear, but 4 week post-induction B-cells and plasma cells are reduced to < 10% of baseline [24]. In addition, 6 months after treatment initiation, 70% of patients exhibit a depletion to ≤ 4 cells/µl [24]. Serum concentrations of glial fibrillary acidic protein (sGFAP) and of neurofilament light chain (sNfL) were significantly lower in patients treated with inebilizumab compared to placebo [6, 7]. ADAs were observed in a subset of participants but had no impact on the efficacy of inebilizumab [24]. The impact of the FCGR3A F/F variant in patients treated with inebilizumab and in the context of B-cell depletion needs to be explored in larger populations [24].

The side effects of inebilizumab treatment include infections and infusion-related reactions, which mainly occur with the first infusion and are mild to moderate in severity [48] (Table 1). No cases of opportunistic infections or reactivations of viral infections have been reported so far. Two patients died during the open-label period: one who had experienced severe pneumonia followed by an attack shortly before enrolment into the open-label period died from respiratory insufficiency shortly after the first dose of 300 mg inebilizumab, while the other developed new neurological symptoms and a large cerebral lesion after three doses of inebilizumab. PML was considered a differential diagnosis but was not confirmed. To date, no other cases of suspected PML or other opportunistic infections associated with inebilizumab therapy have been reported, although a PML warning is included in the product information.

#### Complement inhibition

##### Eculizumab

Eculizumab is a humanized monoclonal antibody that binds to the complement protein C5 and disrupts the terminal complement cascade. It was originally developed for rheumatological diseases and was initially approved in 2007 to treat paroxysmal nocturnal hemoglobinuria (PNH), followed by approval for other indications, including myasthenia gravis. A small pilot study (*n* = 14) [181] followed by the phase III randomized placebo-controlled study (PREVENT) [179, 246] evaluated the efficacy of eculizumab in NMOSD. These studies included only adult AQP4-IgG-positive patients with high disease activity (Table 2). The results of the PREVENT trial showed that eculizumab, as a monotherapy or add-on therapy, significantly reduced the risk of attacks and was effective across all subgroups compared to placebo [172, 180, 246]. Today, eculizumab is approved for the treatment of adult patients with AQP4-IgG-positive NMOSD in the United States (2019), Japan (2019), and other countries. In Europe, it was approved in 2019 for patients with a relapsing disease course (i.e., after a second attack).

Eculizumab has a rapid onset of action and provides continuous near-complete inhibition of C5 activity upon the first infusion. There have been occasional reports of the development of ADAs against eculizumab, but these do not affect the therapeutical efficacy of the drug [69]. An impact of genetic variants in the C5-encoding gene on the response to eculizumab in patients with PNH has been described [161], but their role in NMOSD remains unclear. Long-term effectiveness data for eculizumab in NMOSD are limited, and the OLE was terminated early in most countries after the drug approval [246]. A recent study of 55 patients treated with eculizumab in Germany and Austria reported that all patients were attack-free over a median observation time of 14.6 months (IQR 7.4–21.2) [193].

Long-term safety data for eculizumab use in NMOSD (i.e., treatment > 5 years) are primarily derived from other indications. Serious infections, including those with encapsulated bacteria, such as meningococcal and Neisseria gonorrhoea, have been reported—despite meningococcal vaccination [181, 246]. In the PREVENT trial, one patient receiving concomitant eculizumab and azathioprine treatment died from pleural empyema with positive streptococcal cultures. Infusion-related reactions and anaphylaxis are rare, and no NMOSD patient has discontinued therapy due to these reactions in the phase III trial [179].

##### Ravulizumab

Ravulizumab is a long-lasting monoclonal antibody that targets the complement factor C5. It has been engineered to exhibit altered intracellular antibody recycling and has a four times longer half-life than eculizumab. Therefore, it needs to be administered i. v. only once every 8 weeks. Today, it is approved for the treatment of PNH, aHUS and myasthenia gravis [132], and the EMA recently approved ravulizumab for the treatment of adult patients with AQP4-IgG. Due to its more straightforward application scheme, it may replace the current complement inhibitor eculizumab as in other indications.

The phase III trial investigating ravulizumab for AQP4-IgG-positive NMOSD has met its primary endpoint (CHAMPION–NMOSD) [178]; the OLE is still ongoing (Table 2). In this trial, ravulizumab was given open-label and compared to the placebo arm of the pivotal PREVENT as an external comparator, since a randomized placebo-controlled trial was deemed inappropriate from the perspective of research ethics. Alternatively, a non-inferiority trial comparing ravulizumab with eculizumab was not feasible: Due to the rarity of events (NMOSD attacks), the trial would have necessitated enrolling several thousands of patients.

After at least 50 weeks on drug, no attacks were observed with ravulizumab (compared to 20 in 47 PREVENT placebo patients in the same period of time). No differences in the treatment effect were observed between patients who had received rituximab before ravulizumab compared to those who did not. A sensitivity analysis using propensity score methods revealed that including the historic placebo arm as a comparator did not introduce significant bias [8]. In addition, analysis of ravulizumab’s pharmacodynamics showed a near identical inhibition of C5 as with eculizumab [168]. The onset of action is rapid, and near-complete complement inhibition is observed after the first infusion. The development of ADAs against ravulizumab was so far only observed in patients with myasthenia gravis and not in patients with NMOSD [229]. The side effects of ravulizumab are similar to those of eculizumab. Two meningococcal infections were reported during the CHAMPION trial (one patient was under monotherapy; the other was concomitantly treated with mycophenolate mofetil and prednisolone, had been exposed to rituximab 13 months before and still showed reduced CD19 B-cell counts); both resolved without sequelae. No deaths were reported [178].

Long-term data on ravulizumab are not available yet. Nevertheless, as the study design and efficacy data of both eculizumab and ravulizumab are based on the same comparator (the PREVENT placebo arm), both drugs may be considered closely related members of the same drug class, and recommendations on the use of ravulizumab can reasonably be adopted from the hitherto use of eculizumab.

In general, complement inhibition increases the risk of infections with encapsulated bacteria up to 2000-fold [144]. To prevent such infections, it is essential that patients complete meningococcal vaccination at least 2 weeks before starting on eculizumab or ravulizumab treatment. Alternatively, if therapy needs to be started immediately, such as in many cases of NMOSD, patients must receive antibiotic prophylaxis until at least 2 weeks after completed vaccination. There are no data on the vaccination response in NMOSD patients during or after immunotherapy (e.g., prior rituximab treatment) or shortly after attack treatment (e.g., after high-dose glucocorticoids). Recent reports have shown that NMOSD attacks can occur within 2 weeks of receiving meningococcal vaccination [193], and the European product information on eculizumab warns of disease activity in all eculizumab-treated indications following meningococcal vaccination. As a result, starting treatment with a complement inhibitor under antibiotic prophylaxis and postponing vaccination may be a preferred option. However, more studies are needed to determine the best vaccination timing with an adapted risk–benefit ratio in patients with active NMOSD. As vaccination does not reliably prevent meningococcal infection, patients must be comprehensively counselled about symptoms indicative of meningitis before treatment is started (Table 1).

#### Interleukin-6 receptor blockade

##### Tocilizumab

Retrospective and prospective case series since 2013 have reported that IL-6-R blockade with tocilizumab can effectively prevent attacks in NMOSD, mostly after standard immunotherapies, including rituximab [12, 16, 136, 194, 195]. The recent TANGO trial also showed that tocilizumab is more effective than azathioprine in preventing attacks in highly active NMOSD [255]. However, data on tocilizumab as a first-line therapy for NMOSD are scarce, and tocilizumab has not been granted regulatory approval for NMOSD. The onset of action can be expected after a few weeks. One study investigating tocilizumab treatment within 2 weeks after an NMOSD attack showed favorable effects on the disease course [63]. Nevertheless, data on the long-term use of tocilizumab in NMOSD remain scarce and mainly arise from other indications. Side effects are similar to satralizumab (see next paragraph and Table 1) but include the risk of diverticulitis and gastrointestinal perforations [97].

##### Satralizumab

Satralizumab, a humanized monoclonal IgG2 antibody, has been developed to have optimized antibody recycling and a longer half-life compared to tocilizumab. Two pivotal clinical trials, SAkuraStar and SAkuraSky, have been conducted to evaluate the efficacy of s. c. satralizumab in reducing the attack rate in patients with NMOSD, either as a monotherapy (SAkuraStar) or as an add-on treatment (SAkuraSky; Table 2). Satralizumab was effective in patients with AQP4-IgG-positive but not in patients with AQP4-IgG-negative NMOSD in these trials. However, they did not have sufficient power to draw definitive conclusions for AQP4-IgG-negative patients [223, 250].

Satralizumab has been approved for the treatment of AQP4-IgG-positive NMOSD in adults and adolescents aged 12 and older in Canada (2020), the United States (2020), Japan (2020), South Korea (2021), and Europe (2021). However, there is limited experience using satralizumab as a first-line therapy for adolescents with NMOSD (*n* = 4 in SAkuraStar treated with satralizumab, no adolescents in SAkuraSky). The clinical onset of action for satralizumab is believed to be within 8–12 weeks, based on the results of the two clinical trials [223, 250]. Long-term effectiveness data are available from the OLE of the SAkuraSky and SAkuraStar trials, which included 111 AQP4-IgG-positive patients treated with satralizumab for a median of 4.4 years, and showed that > 70% of patients remained attack-free, 90% were free from severe attacks, and > 85% did not experience worsening of their EDSS score [113]. Long-term safety data from both pivotal trials, collected for up to 7 years with a median treatment exposure of 4 years, have shown no new safety concerns, and no opportunistic infections have been reported thus far [251].

ADAs have been observed in a significant number of patients in the pivotal trials of satralizumab, with rates of 41% and 71%. However, ADAs were found to have no effect on treatment efficacy. Another practical issue is the effect of satralizumab on several CYP450 enzymes, which may require dose adjustments for drugs with a narrow therapeutic index (e.g., warfarin and carbamazepine).

#### Other drugs and interventions

IVIG therapy (“high-dose”; up to 1 g per kg body weight every 4 weeks) may be beneficial in NMOSD and used particularly in children and patients with contraindications to other treatments, or as an add-on therapy. Individual case reports, and case series in AQP4-IgG-positive and -negative NMOSD have shown a reduction of attack frequencies [220, 227]. One study found that IVIG (0.4 g/kg/day every 1–3 months) effectively treated NMOSD as an add-on to azathioprine [130]. Yet, on the other hand, concomitant IVIG administration with monoclonal antibodies may lead to reduced antibody levels. For instance, when given concomitantly with eculizumab, IVIG increased the elimination of eculizumab due to neonatal fragment constant receptor (FcRn) saturation [243]. In addition, IVIG at lower doses (regimens vary from country to country) are substituted in patients who experience symptomatic hypogammaglobulinemia during anti-CD19/20 therapy.

Methotrexate may also be effective either as a monotherapy, a step-down, or an add-on approach [186]. It may be considered as a monotherapy when other drugs are unavailable or contraindicated or for patients with rheumatological comorbidities. In addition, in several retrospective studies, tacrolimus has shown beneficial effects, mainly in Asian NMOSD patients [38, 114].

Finally, intermittent PE or IA combined with or without concomitant immunosuppressive therapy has also been shown to prevent attacks in severe NMOSD in individual case reports [79, 116, 153].

#### Stem cell therapy

Autologous hematopoietic stem cell transplantation (HSCT) has been employed in cases of refractory NMOSD with varying treatment regimens and results, with some improvement in disease activity and disability reported [10, 34, 35, 75, 115]. The inclusion of rituximab in the conditioning regimen before HSCT may improve treatment outcomes. Specifically, administering rituximab twice, once before PE and HSCT and a second time 1 day afterwards, has been suggested to be beneficial [34]. A meta-analysis of 9 studies involving a total of 39 NMOSD patients who underwent autologous HSCT reported a progression-free survival rate of 69%. Another PRISMA-compliant meta-analysis reported a good safety profile with intermediate-intensity regimens [158, 256]. The onset of action of HSCT is prompt, but the procedure bears a risk of severe infections and prolonged immunosuppression, although, unlike allogeneic HSCT, it poses no risk of a graft-versus-host-reaction. Nevertheless, the data on the long-term effectiveness of HSCT in NMOSD remain very limited. Compared to other highly effective approved immunotherapies, HSCT is yet to be studied in randomized, actively controlled trials and—if at all—may be used for individual cases of refractory NMOSD. Other experimental approaches, such as allogeneic HSCT, autologous and allogeneic mesenchymal stem cell transplantation, and peptide-loaded tolerogenic dendritic cell transplantation, have been performed in selected NMOSD patients [115].

#### Ineffective drugs and drugs not recommended

Certain drugs that are approved for MS treatment, such as beta-interferons, glatirameroids, natalizumab, alemtuzumab, sphingosine 1-phosphate receptor modulators, or fumarates, should not be used in patients diagnosed with NMOSD, especially in AQP4-IgG-positive cases. Some agents, such as glatirameroids, are merely ineffective in preventing attacks [17, 174], while others, such as interferon beta, natalizumab, fingolimod, alemtuzumab and dimethyl fumarate, have also been reported to trigger severe attacks [18, 95, 112, 152]. Mitoxantrone may have some effects on NMOSD attack rate [106] but should no longer be used due to its unfavorable safety profile and the limited duration of treatment. Data on cyclophosphamide in NMOSD are conflicting, and its use is not recommended due to the limited total dose allowance and potentially severe side effects [26, 87, 233].

### AQP4-IgG-positive NMOSD: which drug to choose and how to start?

The efficacy of therapeutic antibodies in treating AQP4-IgG-positive NMOSD is superior to classical immunosuppressants and makes them the drugs of choice. Eculizumab, ravulizumab, inebilizumab, rituximab, and satralizumab have all demonstrated efficacy in RCTs for AQP4-IgG-positive NMOSD, although data from eculizumab, ravulizumab, inebilizumab, and satralizumab trials for treatment initiation (“first-line” therapy) after diagnosis is more limited.

Unfortunately, no head-to-head studies between monoclonal antibodies, including rituximab, have been conducted to date. A network meta-analysis on the RCT data of eculizumab, inebilizumab and satralizumab, with time to a first attack as the efficacy outcome, suggests that complement inhibition with eculizumab may be more effective in preventing NMOSD attacks than treatment with inebilizumab or satralizumab [248]. However, these results are limited by several methodological differences across the trials, including study population, inclusion criteria and attack definitions.

Instead, the choice of first-line treatments for NMOSD has to rely on several factors, which include disease activity and severity, mode and onset of action, possibility to combine it with immunosuppressive drugs, effect on autoimmune and other comorbidities, gender (“family planning”), frequency and route of drug administration (intravenous vs. s. c.), side effects and safety profile, as well as drug availability and regulatory approval status (see also Table 3). Factors, such as costs and patient- and physician preferences, also influence treatment decision-making.

**Fig. 2:**
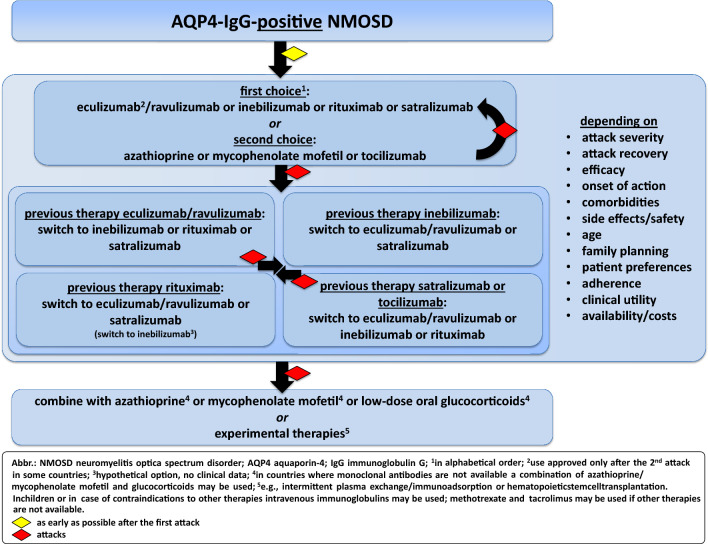
Long-term therapy for AQP4-IgG-positive NMOSD

Age is another important factor to consider. Satralizumab is the only drug that is approved for adolescents (≥ 12 years). Rituximab, azathioprine, and mycophenolate mofetil have been used in NMOSD patients < 18 years; however, data on the treatment of pediatric NMOSD are scarce and beyond the scope of this review. The experience with immunotherapies in very late-onset NMOSD (≥ 70 years) is also limited, and only very few patients in this age group have been studied in the context of RCTs. When choosing immunotherapy in elderly NMOSD patients, immunosenescence and the higher risk of comorbidities and infections should be considered. Some decision-making can be made based on experiences with other diseases, such as myasthenia gravis or rheumatological diseases (Fig. 2).

**Box 4: Recommendation—long-term therapy for AQP4-IgG-positive NMOSD: initiation and selection criteria** **B5** Eculizumab/ravulizumab, inebilizumab, rituximab, satralizumab and tocilizumab are highly effective therapies for AQP4-IgG-positive NMOSD. While there is no high-level evidence demonstrating the superiority of one drug over another, each is suitable for different clinical needs and situations.

**B6** Long-term immunotherapy in AQP4-IgG-positive NMOSD should be initiated with one of the monoclonal antibodies eculizumab/ravulizumab, inebilizumab, rituximab, or satralizumab, whenever those are available and accessible.

**B7** The choice of immunotherapy should be based on factors such as attack severity, attack recovery, efficacy, onset of action, comorbidities, side effects/safety/drug-related mortality, age, family planning, patient preferences, adherence, clinical utility, and availability/costs.

**B8** Long-term immunotherapy with monoclonal antibodies should be started as a monotherapy unless comorbidity warrants a combination with classical immunosuppressive therapies.*

* This does not affect the recommendation of low-dose oral glucocorticoids as oral taper after attack therapy and as bridging therapy when switching immunotherapy—see A2 and B15.

### Does double-negative NMOSD exist, and how shall we treat it?

The existence of true double-negative NMOSD and whether it should be included in diagnostic criteria for NMOSD is currently a matter of debate. The pathology of double- negative NMOSD is less well-understood and many previous studies on AQP4-IgG-negative NMOSD did not test for anti-MOG-IgG as long as MOGAD was not considered a distinct disease and reliable testing was not available. Studies have shown that many patients previously considered seronegative actually harbor in up to 40% MOG-IgG, and the number of true double-negative patients is likely to be lower than previously assumed [46, 77, 91, 109, 187]. In these cases, potential differential diagnoses such as MS, other autoantibody-associated diseases (e.g., anti-GFAP-IgG, anti‐CV2/CRMP5 antibodies), or rare diseases (e.g., neurosarcoidosis) must be thoroughly excluded [66, 88, 93]. If no alternative diagnoses can be found, patients are considered to have double-negative NMOSD. At present, it is unclear if double-negative NMOSD represents a unique disease entity or pathogenetically heterogeneous disease conditions. Thus, previously reported experiences and data on antibody-negative NMOSD should be interpreted with caution.

**Fig. 3:**
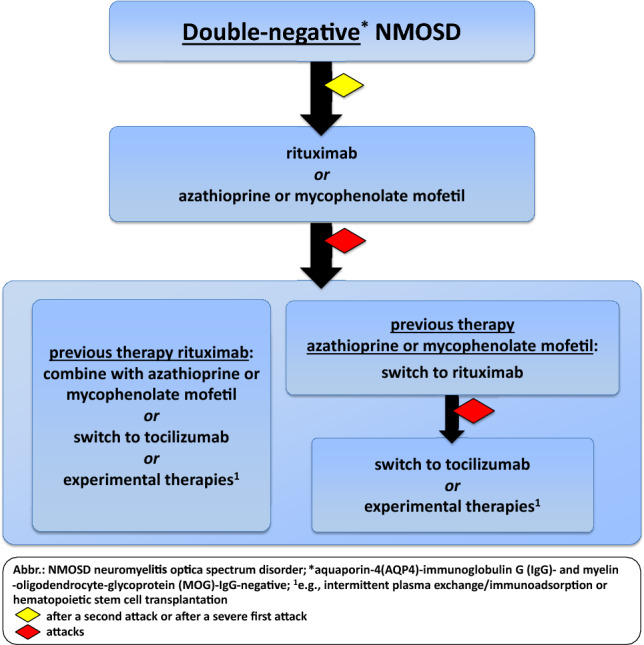
Long-term therapy for double-negative NMOSD

Currently, there are no approved therapies for double-negative NMOSD. Eculizumab, ravulizumab, inebilizumab and satralizumab are approved only for use in AQP4-IgG-positive NMOSD. In the pivotal trials for satralizumab and inebilizumab, some AQP4-IgG-negative NMOSD patients were included (some of them tested positive for MOG-IgG), but the overall number of patients was too low to draw valid conclusions concerning efficacy. Therefore, treatment recommendations for these patients are still based on expert opinions and usually consist of classical immunosuppressive therapies and rituximab. In refractory cases, treatment with tocilizumab has been shown to reduce the risk of attacks in some patients [195, 252, 255]. The observation that attacks in double-negative NMOSD can be severe and result in incomplete remission [56], supports treatment initiation after a first attack. Yet, there are conflicting results regarding the risk of attacks and disability progression in double-negative NMOSD [146, 199, 213] (Fig. 3).

Box 5: Recommendation—long-term therapy: double-negative NMOSD **B9** Research studies should focus on unveiling the significance of “true” double-negative NMOSD, which remains unknown to date.

**B10** Long-term immunotherapy in double-negative NMOSD should be initiated after a second attack or after a severe first attack.

**B11** First-line treatments in double-negative NMOSD are classical immunosuppressive therapies or rituximab, depending on the patient’s characteristics. In case of therapy failure with rituximab, a combination therapy, tocilizumab, or other experimental therapies may be considered.

### Therapy sequence and switching between immunotherapies

Immunotherapies may be switched in case of therapy failure (severe attack during therapy despite of sufficient dosing and sufficient time to expect full action) or due to serious treatment-related side effects or new relevant comorbidities. A clear definition what constitutes a “severe” attack is currently missing, yet, an attack is usually considered severe if a relevant functional deficit or impairment occurs. In addition, the impact of “mild” attacks without relevant functional deficits on the long-term disease course in NMOSD remains unclear and needs further investigation.

Studies on treatment sequences and switching between monoclonal antibodies for NMOSD are limited, with only a few RCTs reporting data on safety aspects. The PREVENT study and the CHAMPION–NMOSD trial both required a 3-month gap between the last administration of rituximab and the initiation of eculizumab/ravulizumab therapy [178, 179], and there is no controlled safety data on shorter intervals. However, a subgroup analysis in the PREVENT study of 26 patients with prior rituximab medication, out of a total of 96 receiving eculizumab, showed a similar safety profile, especially regarding infection rates [172, 246]. As a 3-month gap before starting eculizumab/ravulizumab in patients with therapy failure to one of the antibodies increases the risk of further attacks, prompt initiation of eculizumab/ravulizumab without a defined safety gap to previous therapies seems preferable. Approval studies for other indications have also required similar (atypical hemolytic uremic syndrome) [43] or even longer time intervals (myasthenia gravis) [82] before starting eculizumab. Theoretically, switching from eculizumab to another immunotherapy should not result in long-lasting overlapping effects due to the short half-life of eculizumab of about 12 days [243]. The mean terminal elimination half-life of ravulizumab is longer and around 50 days. The use of rituximab shortly after or concomitantly with eculizumab has only been described in single case reports for patients with other conditions; other data on therapies with monoclonal antibodies after eculizumab or ravulizumab are not available [157, 191]. Eculizumab inhibits the complement-dependent cytotoxic effects of rituximab and thus can reduce the expected pharmacological effects of rituximab [58]. The subsequent therapy's onset and mode of action should be considered to prevent disease activity from reoccurring after stopping eculizumab/ravulizumab, and a short time interval is preferred. Bridging with oral glucocorticoids may also be considered [181].

Regarding inebilizumab, the initial phase I study in MS required a 12-month gap from any previous monoclonal antibody medication [3]. The N-MOmentum trial required a 6-month gap to rituximab and a 3-month gap to eculizumab or tocilizumab, but this study included only a small number of patients who had undergone these previous therapies [51]. The trial included 17 patients with a history of rituximab therapy, and among them, 13 were randomized to inebilizumab. Seven of them had a previous history of attacks during rituximab therapy, and none of them had an attack during inebilizumab treatment [68], but it remains unclear whether switching from anti-CD20 therapy to anti-CD19 therapy in case of disease activity is rational. Overall, 16 participants (94%) with prior use of rituximab experienced infections, compared to 70% without prior use of rituximab, and a greater proportion of patients with prior rituximab had IgG levels of < 500 mg/dL. There is no data on switching from inebilizumab to another monoclonal antibody. The half-life of inebilizumab is around 18 days, and B-cell depletion below the lower limit of normal is maintained in 94% of patients for at least 6 months following treatment. The exact time for B-cell repletion is not known. Extrapolation from the rituximab data should be handled with care as inebilizumab depletes a broader range of cells.

Trials with IL6-R blockers have similar time gaps to prior antibody medication. The TANGO trial showed a good safety profile for tocilizumab use more than 6 months after rituximab therapy [255]. A case series including three patients receiving rituximab up until < 6 months before and one patient right until the initiation of tocilizumab reported no severe infections [194]. The SAkura trials required a gap of 6 months from prior rituximab or eculizumab use [223, 250]. The ongoing SAkuraBONSAI trial is investigating the efficacy and safety of satralizumab in patients treated unsuccessfully with rituximab within the last 6 months [222]. From other indications, there are no reported infectious complications after the immediate switch from rituximab to tocilizumab, but some patients may develop neutropenia grade 3 [123, 195]. Data on patients with rheumatoid arthritis (*n* = 27) with tocilizumab to rituximab sequential therapy without a gap revealed no new safety signals after switching to rituximab [62]. The half-life of tocilizumab is 8–18 days depending on the dosage and route of administration. Due to the prolonged terminal half-life of satralizumab (approximately 30 days), the effect of satralizumab may persist for several weeks after stopping treatment. So far, no published data exist on switching from satralizumab to eculizumab, inebilizumab or rituximab.

In general, it is essential to be aware of the potential risk factors for severe infections with prior immunosuppressive treatments, pre-existing low IgG levels, older age, and concomitant glucocorticoid therapy [215]. The ongoing risks while continuing therapy must be weighed against the risk of re-occurring disease activity in case of cessation. In individual cases, long-lasting therapies (B-cell-directed therapies) may be switched to short-lasting therapy. Whether patients should switch between monoclonal antibody therapies due to potential side effects and whether a “prophylactic” switch may prevent severe side effects remains unanswered. For example, it remains unclear whether patients should switch from B-cell therapies to another monoclonal antibody or reduce dosage in case of increasing and severe hypogammaglobulinemia before experiencing relevant infections. Currently, there is a lack of data to support either approach.

Box 6: Recommendation—long-term therapy: switching drugs **B12** In case of treatment failure with classical immunosuppressive therapies, therapy should be switched to a monoclonal antibody.

**B13** In case of treatment failure with a monoclonal antibody, therapy should be switched to another monoclonal antibody, preferably with a different mode of action.

**B14** The interval between therapies should be as short as possible and based on the mode and latency to onset of action of the subsequent therapy, as well as potential side effects resulting from overlapping treatments.

**B15** When switching immunotherapy, bridging therapy with low-dose oral glucocorticoids should be performed for up to 3–6 months, depending on the mode and onset of action of the subsequent therapy, duration of action of the previous therapy, disease activity, comorbidities, and side effects.

### Combination therapies

Except for inebilizumab, other monoclonal antibody therapies (eculizumab, ravulizumab, satralizumab, rituximab) for NMOSD have been evaluated in combination with immunosuppressive therapies, including azathioprine, mycophenolate mofetil and oral glucocorticoids. Thus far, studies have not shown a clear benefit in using combination therapies (i.e., antibody therapy combined with immunosuppressive therapies) over monotherapy with monoclonal antibodies for NMOSD. For patients suffering exclusively from NMOSD, monotherapy with monoclonal antibodies may be favored as the first-line treatment, as there is a risk of additional adverse events with combination therapy. However, immunosuppressive therapy may be offered as add-on therapy to patients refractory after treatment with > 2 monoclonal antibodies in succession—or in countries where alternative monoclonal antibodies are not available. Immunosuppressive drugs may also be added if necessary to treat comorbidities, such as SLE and Sjögren’s syndrome. In countries where monoclonal antibodies are not available at all, a combination of azathioprine/mycophenolate mofetil and glucocorticoids may be used. Inebilizumab has not been tested in combination with other immunosuppressants and is approved by the European Medicines Agency only as a monotherapy. If it is combined with another immunosuppressive therapy, the potential for increased immunosuppressive effects and side effects should be considered.

On the other hand, in patients with breakthrough disease during a course of classical immunosuppressive drugs, it is recommended to switch to antibody therapy immediately and continue immunosuppressive treatment at least temporarily or to bridge with oral glucocorticoids, to minimize the risk of a (severe) attack during the transition. Nevertheless, due to a lack of data, it remains a matter of debate when and how to stop background immunosuppressive therapies in patients who stabilize after starting antibody therapy. Data from the OLE of eculizumab showed that 37% of patients were able to stop or decrease their immunosuppressive therapies and remain stable [246], while data from the OLE of the SAkuraSky trial evaluating satralizumab in combination with immunosuppressive therapies showed that 16 out of 36 patients were able to taper their glucocorticoid dose, 3 stopped it entirely and remained attack-free, while 2 experienced three attacks during tapering [249]. Interactions between the various treatment modalities need to be considered. As mentioned above, for example co-treatment with IVIG can diminish the efficacy of eculizumab.

### Treatment monitoring in NMOSD

All medications, including off-label and approved therapies, carry potential risks, and long-term post-marketing experience for registered drugs is limited. Therefore, thorough monitoring and patient education are necessary to minimize risks. Infectious risks may be increased during immunosuppression, and the risk may accumulate with the duration of treatment. Older patients likely are at higher risk due to immunosenescence and the occurrence of comorbidities [78, 151, 204]. Different therapies alter the patient’s immune system and immune cells to a variable extent, hence specific monitoring is required. Potential therapy-related complications, including gastrointestinal side effects, liver toxicity, as well as cardiovascular and cancer risks, should be considered (see Table 3). Practical monitoring recommendations also include performing brain and spinal cord MRI for safety surveillance and to reassure the diagnosis during the initial phase of the disease, at the time of acute attacks, at the therapy switch, and in the case of a suspected CNS infection (e.g., PML).

It is essential to continuously monitor the disease activity to detect treatment failure through therapy management and regular follow-up visits. Treatment failure is mainly defined as the occurrence of a new attack (see above severe vs. mild attack). The NEDA concept of “No Evidence of Disease Activity” applied in patients with MS is not established in NMOSD yet, and the significance of asymptomatic brain and spinal cord lesions regarding long-term disability remains unclear. In addition, the relevance of neurodegeneration (brain and spinal cord atrophy as well as retinal fiber loss) in the context of immunotherapies needs to be further investigated. Structured neurological examinations (including EDSS and FSS since generally accepted NMOSD-specific scales do not exist thus far) should be performed at the beginning and switch of therapy, in case new symptoms occur and, in the absence of clinically apparent attacks, every 6–12 months. Additional tests and scores, such as opticospinal impairment score, Hauser Ambulation Index, 9-Hole-Peg-Test (9HPT), Timed-25-Foot-Walk (T25FW) as well as quality of life measures may be used. More recently, a strong association between EDSS and quality of life dimensions measured by EuroQol 5 has been demonstrated [126]. Evoked potentials, MRI, and optical coherence tomography (OCT) can also be used to help monitor the long-term evolution of atrophy [36, 40, 117, 162–164, 169] and distinguish attacks from pseudo-attacks [100, 197].

There is no clear correlation between AQP4-IgG titers and disease activity, and monitoring of antibody levels for attack prediction is, therefore, not recommended in general [87, 133, 203]. However, some patients may become at least temporarily antibody-negative with B-cell-directed therapies or anti-IL-6-R therapy over time. Recently one large study (933 initially AQP4-gG positive patients) demonstrated that seroreversion is rare (11%), occurs predominantly in younger patients (age < 20 years) and in patients with initially low AQP4-IgG titers and is often transient [139]. Patients who serorevert are still considered AQP4-IgG-positive NMOSD cases, as in the RIN-1 trial. Overall the significance of transient as well as sustained seroreversion is currently unknown but testing antibody levels at least every 1–2 years could help increase knowledge on this topic and aid in future therapy guidance.

### Duration of therapy

Long-term immunotherapy for NMOSD is associated with a potentially increased risk for side effects, especially for adolescents and young adults who may be on therapy for decades. There is limited data on the cessation or “de-escalation” of immunosuppressive therapies in NMOSD. A recent French study showed that rituximab de-escalation (including increased infusion intervals or switching to oral therapies) and discontinuation in AQP4-IgG-positive and double-negative NMOSD patients is associated with an increased risk for attacks in the following 12 months [55]. One retrospective study from Korea found that 14 out of 17 AQP4-IgG-positive NMOSD patients (82%) suffered an attack at a median interval of 6 months after discontinuation of immunosuppressive therapies (including azathioprine, mycophenolate mofetil, rituximab), despite being stable beforehand for a median of 5 years [104]. In that study, 5 (29%) were AQP4-IgG-negative at the time of discontinuation, but 4 (80%) of them reverted from AQP4-IgG-negative to AQP4-IgG-positive after treatment cessation. Another study reported a high attack ratio (77.5%; n = 100) after discontinuing immunosuppressive drugs (azathioprine > mycophenolate mofetil > rituximab), with a higher rate in patients with a previous history of longitudinally extending transverse myelitis (LETM) or shorter disease duration. Reasons for discontinuation included the patient’s decision, side effects, and desire for pregnancy [129]. In another small case series (*n* = 4; 3 adolescents), two patients remained stable after ceasing rituximab treatment, one turned AQP4-IgG-negative and showed no further attack for up to 10.5 years, and two experienced attacks after 3 and 5 years, respectively [239]. As a single attack can result in significant disability, stopping immunotherapy is currently not recommended in AQP4-IgG-positive patients. Data on treatment discontinuation in double-negative NMOSD is scarce, making it even more challenging to provide recommendations on treatment duration. However, accounting for the more heterogeneous aetiology and mixed prognosis of this group of patients, stopping therapy may be an option in those who were stable for at least 5 years and have had only few and mild attacks.

Serum GFAP levels have been found to be indicative of disease activity in AQP4-IgG-positive NMOSD and are positively correlated with the EDSS [7, 101]. However, further research is needed to determine the utility of serum GFAP levels as a biomarker for assessing attack risk and monitoring in AQP4-IgG-positive or seroconverted patients who discontinue therapy [202].

Box 7: Recommendation—long-term therapy: duration **B16** Immunotherapy should be continued in stable AQP4-IgG-positive NMOSD patients and patients must be closely monitored if treatment is temporarily or permanently discontinued due to side effects or patient choice. In double-negative NMOSD patients who have been stable for over 5 years, re-evaluation of immunotherapy may be considered (expert opinion).

**B17** Research studies should focus on investigating the significance of seroreversion to seronegativity, which remains unknown to date.

### Pregnancy and NMOSD

NMOSD primarily affects women, with a male-to-female ratio among AQP4-IgG-positive patients of approximately 1:10 [31, 92]. AQP4 is highly expressed in the human placenta, and women with active disease have an increased risk for spontaneous abortions and eclampsia [189, 190, 201, 208]. Although AQP4-IgG can pass the placenta, it has not been shown to have a pathogenic effect on the newborn in the few cases reported thus far [196].

In 2020, therapeutic considerations on pregnancy-related treatment issues in NMOSD were published [140], and more recently, a detailed review and consensus-based recommendations on pregnancy and NMOSD have been proposed by the French MS society [230]. For detailed information on drugs used for the treatment of NMOSD and related risks, we refer to those reviews. Pregnancy is associated with an increased risk of attacks postpartum, potentially also during the third trimester of pregnancy [140]. Factors that increase the risk of disease activity during pregnancy and/or postpartum include a positive AQP4-IgG serostatus, young age, disease activity before pregnancy, and the withdrawal of immunotherapy. There have been numerous reports of attacks occurring in patients who stopped immunotherapy in preparation for pregnancy [44, 57, 102, 120, 234]. Therefore, it is important for women with NMOSD to plan their pregnancy and be advised ahead of time carefully.

Medications, such as mycophenolate mofetil and methotrexate, which are teratogenic, must be discontinued before pregnancy (Table 3). By contrast, in one study (*N* = 81), treatment with rituximab shortly prior to pregnancy has been found to reduce the attack risk without causing severe side effects in newborns [120]. Therapy with rituximab may offer the advantage of a continued long-lasting effect during pregnancy. In general, IgG1 monoclonal antibodies do not cross the foetoplacental barrier in the first trimester but increasingly transfer in the second and third trimesters [72]. In case rituximab is administered during pregnancy due to disease activity, patients must be counseled and advised that data on its safety and efficacy in pregnant women with NMOSD are limited. Importantly, in babies of mothers treated with B-cell therapies, live or attenuated live vaccines (but not non-live vaccines) should be given only after repletion of B cells. If infusions are paused during pregnancy, they should be restarted soon after delivery.

Another treatment option considered relatively safe for pregnant women with NMOSD is azathioprine, although recent meta-analyses and a retrospective cohort study on women with inflammatory bowel disease reported an increased risk of preterm birth and a twofold increased rate for stillbirth (1% vs. 0.5% in untreated) [150, 221, 257]. Female patients with a stable disease during azathioprine therapy who become pregnant should continue treatment but must be thoroughly counseled on potential risks.

Eculizumab exposure during pregnancy was found to be safe in a small retrospective registry study (*n* = 75) of women with atypical hemolytic uremic syndrome. Another observational study of 24 pregnancies in women with PNH showed that eculizumab exposure during pregnancy resulted in 85% live births, with no observed malformations [99, 200]. Female patients who are pregnant or planning to become pregnant while receiving eculizumab or ravulizumab may continue therapy after carefully weighing risks and benefits, although data on its safety and efficacy in pregnant women with NMOSD are limited. Tocilizumab has been used during pregnancy in women with rheumatoid arthritis, and no increased risk of malformations has been reported thus far, although the risk for spontaneous abortion and preterm birth may be slightly elevated [80, 238]. Data on pregnancy outcomes under satralizumab and inebilizumab are very limited and have not yet been published. Female patients who are pregnant or are planning to become pregnant while receiving immunotherapy with IL-6-R blockade may continue the therapy if the assumed benefits clearly outweigh the risks, but the potential risk of spontaneous abortion or preterm birth and the paucity of data must be discussed with the patient.

Oral glucocorticoids may increase the risk of cleft palate formation if administered during the first trimester and have also been associated with growth retardation and premature birth. Therefore, they should only be used after a careful risk–benefit assessment [140]. Fluorinated glucocorticoids (e.g., dexamethasone) should be avoided during the entire pregnancy, since they clearly increase the risk of congenital anomalies, intrauterine growth retardation and behavioural disorders in the offspring.

Treatment options for attacks during pregnancy include high-dose glucocorticoids and apheresis therapy (preferably with IA). The choice of treatment will depend on the severity of the attack and the stage of gestation [118]. Although glucocorticoids can transfer to breastmilk, they can be administered during breastfeeding as the infant’s exposure to the drug is minimal and even lower if breastfeeding is delayed for 2–4 h after glucocorticoid infusion [32].

The transfer of drugs into breast milk is influenced by various factors, including the molecule size and lipophilicity, as well as the stage of the breast milk [231]. For instance, the amount of drug present in the colostrum is generally higher than in mature milk. Methotrexate and mycophenolate mofetil should not be given during breastfeeding. Monoclonal antibodies are a viable option during breastfeeding as the amount that transfers into the mature breastmilk is minimal, and when ingested orally by the newborn, the pharmacologically effective amount of antibody in the baby’s serum is further minimized [42, 119, 121]. Although data are limited, there is some experience with rituximab and eculizumab during breastfeeding, suggesting that treatment with rituximab and eculizumab during lactation might be safe [42, 99]. In general, an interdisciplinary approach involving neonatologists and gynaecologists is important when considering therapies during pregnancy and postpartum.

Limited data are available on the effects of NMOSD on fertility. AQP4-IgG may potentially affect fertility as AQP4 is expressed in the paraventricular hypothalamus [9, 226]. Azathioprine and monoclonal antibodies appear to have only minimal effects on fertility. However, drugs such as methotrexate and mitoxantrone have been shown to decrease fertility in women and affect ovarian reserve and sperm count [140].

Box 8: Recommendation—long-term therapy: family planning and pregnancy **B18** Female patients of reproductive age with AQP4-IgG-positive NMOSD must be counseled early on regarding family planning options and the risks and benefits of both pregnancy and immunotherapies during pregnancy.

**B19** Pregnancy should be planned during a stable phase of the disease.

**B20** Teratogenic drugs such as mycophenolate mofetil or methotrexate should be avoided in patients of child-bearing age and must be replaced with safer options prior to pregnancy.

**B21** Long-term immunotherapy should not be discontinued or postponed for the desire to become pregnant. Monoclonal antibodies (eculizumab/ravulizumab, rituximab, tocilizumab)* or azathioprine should be continued during pregnancy. The decision which of these drugs to use during pregnancy should be based on factors such as the drug’s half-life, duration of action, risk of disease reoccurrence after cessation, risk/benefit ratio, patient preference, and availability.

**B22** Female patients who continue therapy during pregnancy must be thoroughly counseled on potential risks, including infections. A strict risk/benefit assessment must be performed before pregnancy and a close monitoring of mother and fetus throughout the pregnancy and postpartum.

**B23** Given the limited data on the use of monoclonal antibodies during pregnancy, rituximab should be preferred for female patients who are planning to become pregnant in the near future.

**B24** Female patients with a stable disease under azathioprine who become pregnant should continue treatment.

**B25** If exposure to anti-B-cell-directed drugs occurs during pregnancy, lymphocyte and B-cell count testing in the newborn (umbilical cord blood) should be performed.

**B26** If monoclonal antibodies are continued during pregnancy, the timing of live attenuated vaccinations must be discussed with pediatricians and carefully planned.

**B27** In case of treatment interruption during pregnancy, long-term immunotherapy should be resumed shortly after delivery.

*Based on currently available data.

### What role do vaccinations play?

There have been several case reports of first occurrences and flare-ups of NMOSD in temporal association with vaccinations, including COVID-19 vaccines [60, 85, 86, 145, 211]. However, no causal link could be established so far. Moreover, infections (including COVID-19) may contribute to the development of NMOSD and often precede NMOSD attacks, rendering vaccinations potentially important in NMOSD [258]. Furthermore, vaccinations are an essential element in increasing the safety of immunotherapeutic treatments for NMOSD. For example, meningococcal vaccination and/or antibiotic prophylaxis are required before starting eculizumab therapy and should be repeated at least every 2–3 years [143]. It is also important to check vaccination status before starting B-cell-directed therapies, as these treatments may reduce the humoral immune response [11, 167]. Due to the risk of hepatitis B reactivation and severe hepatitis B infection during B-cell-directed therapies, screening for anti-HBV titers should be performed, and vaccinations should be updated accordingly [13, 177]. At the same time, postponing immunotherapy due to an incomplete vaccination status bears the risk of attacks during the vaccination period [193]. Moreover, the impact of prior or ongoing immunotherapies on vaccination responses varies. In general, it is important to update all vaccinations according to national guidelines in a standard and timely manner, considering individual timing, disease activity, the benefits of vaccinations and the fact that (attenuated) live vaccines must not be used in immunosuppressed individuals.

Box 9: Recommendation—vaccinations **B28** In patients with active NMOSD, therapy initiation must not be postponed due to incomplete vaccination status. Vaccinations should be updated according to national recommendations and standards as soon as possible.

**B29** Research studies should focus on evaluating the impact of vaccinations on the disease course and vaccination response during long-term immunotherapy in NMOSD patients.

### Future therapies

Chimeric antigen receptor (CAR) T-cell therapy targeting B-cell antigens, such as CD19, is being investigated for autoimmune diseases, including NMOSD [2]. A phase I trial of CAR T-cell therapy targeting B-cell maturation antigen (BCMA) in 12 AQP4-IgG-positive NMOSD patients showed no attacks in 11 patients during a follow-up of 5.5 months (median), with hematologic toxic side effects and cytokine release syndrome being reported as potential side effects [184].

Other new drugs and new B-cell- or plasma-cell-directed therapies, such as ublituximab, T4406F telitacicept (B-lymphocyte stimulator blocker and proliferation-inducing ligand), bortezomib (proteasome inhibitor) and Bruton’s tyrosine kinase inhibitor SHR1459 [59, 147, 254] (clinicaltrials.gov), are being actively investigated for the prevention of attacks in AQP4-IgG-seropositive NMOSD. FcRn-inhibiting agents, which have shown efficacy in treating antibody-mediated autoimmune diseases, are also being investigated as potential treatments for NMOSD [71, 81]. The antihistamine drug cetirizine, which blocks eosinophils, has shown promising results as an add-on to other immunotherapies in a pilot trial [98].

Approaches to restoring immune tolerance and potentially inducing permanent remission in NMOSD include DNA or T-cell vaccination and the administration of AQP4 peptide-loaded autologous tolerogenic dendritic cells [21, 212, 259]. Aquaporumab, an antibody against AQP4, has been shown to have beneficial effects in a NMOSD mouse model but has not yet been tested in human trials [64, 224]. Such treatments might turn out to be beneficial for AQP4-IgG-positive patients by stopping disease activity and promoting tissue repair, potentially helping patients to avoid chronic long-term immunosuppressive therapies. However, reliable evidence is missing thus far.

### Conclusions

Recent insights into the pathogenesis of NMOSD have led to the development of novel targeted and highly effective therapies for patients with AQP4-IgG-positive NMOSD. These therapies provide a more personalized approach to treatment, considering factors, such as disease activity, age, comorbidities, family planning, side effects, route of administration, patient choice, availability, and costs. They also allow for switching between therapies in case of side effects or insufficient treatment response. However, long-term experience and therapy sequences, as well as general risk management for potential lifelong therapies, are still being developed. To gather these “real-world” data, registries and platform trials should be established, and a standardized approach to data collection should be adopted.

Unmet needs in NMOSD therapy include understanding the long-term disease course, determining optimal immunotherapy durations, developing strategies for treatment cessation and de-escalation, and searching for biomarkers indicating attack risk. Double-negative NMOSD, which affects a minority of patients, should also be addressed in international and collaborative research. Finally, NMOSD is a global disease, and patients and caregivers worldwide should have equal and affordable access to available therapies and therapeutic knowledge.

### Supplementary Information

Below is the link to the electronic supplementary material.

Supplementary file1 (DOCX 21 KB)

## Abbreviations

ADA: Anti-drug antibodies
AQP4: Aquaporin-4
AQP4-IgG: Aquaporin-4 immunoglobulin G
CNS: Central nervous system
CSF: Cerebrospinal fluid
EDSS: Expanded Disability Status Scale
FCGR3A: Fragment c gamma receptor 3A
FSS: Functional system scores
GFAP: Glial fibrillary acidic protein
HSCT: Hematopoietic stem cell transplantation
HBV: Hepatitis B virus
IA: Immunoadsorption
IL-6: Interleukin-6
IL-6-R: Interleukin-6 receptor
i. v.: Intravenously
IVIG: Intravenous immunoglobulins
MRI: Magnetic resonance imaging
MP: Methylprednisolone
MOG: Myelin–oligodendrocyte–glycoprotein
MOGAD: MOG-IgG-associated disease
MS: Multiple sclerosis
FcRn: Neonatal fragment constant receptor
NfL: Neurofilament light chain
NMOSD: Neuromyelitis optica spectrum disorder
NEMOS: Neuromyelitis Optica Study Group
OLE: Open-label extension
PNH: Paroxysmal nocturnal hemoglobinuria
PE: Plasma exchange
PML: Progressive multifocal leukenzephalopathy
RCT: Randomized controlled trial
SLE: Systemic lupus erythematosus
s. c.: Subcutaneous
T25FW: Timed-25-Foot-Walk
9HPT: 9-Hole-Peg-Test
