# Correction to: Update on the diagnosis and treatment of neuromyelitis optica spectrum disorders (NMOSD) – revised recommendations of the Neuromyelitis Optica Study Group (NEMOS). Part II: Attack therapy and long-term management

**DOI:** 10.1007/s00415-024-12288-2

**Published:** 2024-04-05

**Authors:** Tania Kümpfel, Katrin Giglhuber, Orhan Aktas, Ilya Ayzenberg, Judith Bellmann-Strobl, Vivien Häußler, Joachim Havla, Kerstin Hellwig, Martin W. Hümmert, Sven Jarius, Ingo Kleiter, Luisa Klotz, Markus Krumbholz, Friedemann Paul, Marius Ringelstein, Klemens Ruprecht, Makbule Senel, Jan-Patrick Stellmann, Florian Then Bergh, Corinna Trebst, Hayrettin Tumani, Clemens Warnke, Brigitte Wildemann, Achim Berthele, The Neuromyelitis Optica Study Group (NEMOS), Philipp Albrecht, Klemens Angstwurm, Susanna Asseyer, Ana Beatriz Ayroza Galvao Ribeiro Gomes, Antonios Bayas, Stefanie Behnke, Stefan Bittner, Franziska Buetow, Mathias Buttmann, Ankelien Duchow, Daniel Engels, Thorleif Etgen, Katinka Fischer, Benedikt Frank, Anna Gahlen, Achim Gass, Johannes Gehring, Christian Geis, Ralf Gold, Yasemin Göreci, Jonas Graf, Sergiu Groppa, Matthias Grothe, Julia Gutbrod, Kersten Guthke, Axel Haarmann, Maria Hastermann, Bernhard Hemmer, Mariella Herfurth, Marina Herwerth, Frank Hoffmann, Olaf Hoffmann, Martin W Hümmert, Leila Husseini, Jutta Junghans, Matthias Kaste, Peter Kern, Karsten Kern, Pawel Kermer, Christoph Kleinschnitz, Wolfgang Köhler, Kimberly Körbel, Markus Kowarik, Markus Kraemer, Julian Kretschmer, Natalia Kurka, Theodoros Ladopoulus, Ann-Sophie Lauenstein, Sarah Laurent, De-Hyung Lee, Dominik Lehrieder, Frank Leypoldt, Martin Liebetrau, Ralf Linker, Gero Lindenblatt, Lisa Lohmann, Felix Lüssi, Peter Luedemann, Michelle Maiworm, Martin Marziniak, Christoph Mayer, Stefanie Meister, Mathias Mering, Imke Metz, Sven Meuth, Jasmin Naumann, Oliver Neuhaus, Tradite Neziraj, Moritz Niederschweiberer, Sabine Niehaus, Carolin Otto, Florence Pache, Thivya Pakeerathan, Sarah Passoke, Marc Pawlitzki, Hannah Pellkofer, Mosche Pompsch, Anne-Katrin Pröbstel, Refik Pul, Sebastian Rauer, Nele Retzlaff, Arne Riedlinger, Paulus Rommer, Veith Rothhammer, Kevin Rostásy, Rebekka Rust, Christoph Ruschil, Matthias Schwab, Maria Seipelt, Patrick Schindler, Carolin Schwake, Patricia Schwarz, Claudia Sommer, Alexander Stefanou, Till Sprenger, Andreas Steinbrecher, Heike Stephanik, Muriel Stoppe, Klarissa Stürner, Marie Süße, Athanasios Tarampanis, Simone Tauber, Daria Tkachenko, Annette Walter, Klaus-Peter Wandinger, Anna Walz, Martin Weber, Jens Weise, Jonathan Wickel, Heinz Wiendl, Alexander Winkelmann, Yavor Yalachkov, Uwe Zettl, Ulf Ziemann, Frauke Zipp

**Affiliations:** 1 Institute of Clinical Neuroimmunology, LMU Hospital, Ludwig-Maximilians-Universität München, Munich, Germany; 2 Department of Neurology, School of Medicine, Technical University Munich, Klinikum Rechts der Isar, Munich, Germany; 3 Department of Neurology, Medical Faculty, Heinrich Heine University Düsseldorf, Düsseldorf, Germany; 4 Department of Neurology, St. Josef Hospital, Ruhr University Bochum, Bochum, Germany; 5 Department of Neurology, Charité—Universitätsmedizin Berlin, corporate member of Freie Universität Berlin and Humboldt-Universität zu Berlin, Berlin, Germany; 6 Experimental and Clinical Research Center, a cooperation between the Max Delbrück Center for Molecular Medicine in the Helmholtz Association and Charité—Universitätsmedizin Berlin, Berlin, Germany; 7 Max Delbrück Center for Molecular Medicine in the Helmholtz Association (MDC), Berlin, Germany; 8 NeuroCure Clinical Research Center, Charité Universitätsmedizin Berlin, corporate member of Freie Universität Berlin and Humboldt-Universität Zu Berlin, and Berlin Institute of Health, and Max Delbrück Center for Molecular Medicine, Berlin, Germany; 9 Department of Neurology and Institute of Neuroimmunology and MS (INIMS), University Medical Center Hamburg-Eppendorf, Hamburg, Germany; 10 Department of Neurology, Hannover Medical School, Hannover, Germany; 11 Molecular Neuroimmunology Group, Department of Neurology, University of Heidelberg, Heidelberg, Germany; 12 Marianne-Strauß-Klinik, Behandlungszentrum Kempfenhausen für Multiple Sklerose Kranke, Berg, Germany; 13 Department of Neurology with Institute of Translational Neurology, University of Münster, Münster, Germany; 14 Department of Neurology and Pain Treatment, Immanuel Klinik Rüdersdorf, University Hospital of the Brandenburg Medical School Theodor Fontane, Rüdersdorf bei Berlin, Germany; 15 Faculty of Health Sciences Brandenburg, Brandenburg Medical School Theodor Fontane, Rüdersdorf bei Berlin, Germany; 16 Department of Neurology & Stroke, University Hospital of Tübingen, Tübingen, Germany; 17 Department of Neurology, Center for Neurology and Neuropsychiatry, LVR-Klinikum, Heinrich Heine University Düsseldorf, Düsseldorf, Germany; 18 Department of Neurology, University of Ulm, Ulm, Germany; 19 APHM, Hopital de la Timone, CEMEREM, Marseille, France; 20 Aix Marseille University, CNRS, CRMBM, Marseille, France; 21 Department of Neurology, University of Leipzig, Leipzig, Germany; 22 Department of Neurology, Faculty of Medicine, University Hospital Cologne, University of Cologne, Cologne, Germany

**Correction to: Journal of Neurology** 10.1007/s00415-023-11910-z.

In the original version of this article, mistakes were noticed in Table 1 and Table 2.

In the Table 1, text in the column “Application” and row “Ravulizumab” which previously read:

i. v., weight-based^2^, loading dose of 2400–3000 mg on days 1 and 15 followed by 3000–3600 mg once every 8 weeks.

Should have read:

i. v., weight-based^2^, loading dose of 2400–3000 mg on day 1 followed by the maintenance dose (3000–3600 mg) after 2 weeks and then once every 8 weeks.

In the Table 2, text in the column “Inclusion criteria” and row “Ravulizumab” which previously read:

 ≥ 2 attacks in 12 months or ≥ 3 attacks in 24 months with 1 attack in the last 12 months.

Should have read:

 ≥ 1 relapse in the last 12 months.

The corrected Tables 1 and 2 are given in the following page:

**Table 1:** Recommendations on the application, monitoring, and important risks of preventive immunotherapies in NMOSD^1,2^

Drug	Application	Full onset of action	Common side effects	Risk of infections	Other important risks	Blood monitoring	Additional suggested monitoring
Azathioprine	Oral, daily, 2.5–3.0 mg/kg body weight	After 6–12 months	Hematological abnormalities (lymphocytopenia, pancytopenia), elevation of liver enzymes, gastrointestinal side effects	Upper respiratory tract and urinary tract infections, opportunistic infections	Drug-induced fever, several drug interactions (including allopurinol, anti-viral and anti-coagulatory drugs), photosensitization (skin), increased cancer risk with duration of treatment (> 10 years)	BCC and differential WBCC, liver enzymes	Regular screening for cancer (by dermatologist, gynecologist)
Mycophenolate mofetil	Oral, daily, 1000–2000 mg	After 8–12 weeks	Hematological abnormalities (anemia), elevation in liver enzymes, gastrointestinal side effects	Upper respiratory tract and urinary tract infections, opportunistic infections	Increased cancer risk, teratogenic and embryotoxic effects	BCC and differential WBCC, liver enzymes	Regular screening for cancer (by dermatologist, gynecologist)
Glucocorticoids	For attack: i. v., 1x/day, 1000–2000 mg, over 3–5 days For taper: oral, 1x/day, starting with 1 mg/kg/day or 20–30 mg/day and then tapered to 10–15 mg within 2–3 weeks; For long-term use: oral, 1x/day, individual dosing (ideally ≤ 7.5 mg/day)	Immediate effects	Hematological abnormalities (lymphopenia), elevation in liver enzymes	Increased risk for infections, including opportunistic infections (e.g., PJP, particularly if administered with other IS)	Diabetes, arterial hypertension, osteoporosis, cataract, adrenal insufficiency, Cushing’s syndrome	BCC and differential WBCC, liver enzymes, electrolytes, blood glucose	Use of glucocorticoids in combination with proton pump inhibition and thrombosis prophylaxis Monitoring of blood pressure; with long-term use: bone densitometry, eye examination, cardiovascular check-ups
Rituximab	i. v., with premedication (glucocorticoids, antiphlogistics, antihistamines); usually initially 1000 mg at day 1 and day 14, followed by 500–1000 mg every 6 months	B-cell depletion within 4 weeks, full onset of action after 8–12 weeks	Nausea, exanthema, headache	Upper respiratory tract and urinary tract infections, hepatitis B reactivation, opportunistic infections (including PML), no PML in NMOSD reported so far	Infusion-related pseudo-allergic reactions (due to cell lysis), leucopenia, neutropenia and LON, hypogammaglobulinemia	Differential WBCC, serum immunoglobulins, CD19/20-positive B-cell count	Monitoring for infusion-related (allergic) reactions
Inebilizumab	i. v., with premedication (glucocorticoids, antiphlogistics, antihistamines); initially 300 mg at day 1 and day 14, followed by 300 mg every 6 months	B-cell depletion within 2 weeks, full onset of action after 6–8 weeks	Arthralgias, back pain	Upper respiratory tract and urinary tract infections, opportunistic infections (including PML)	Infusion-related pseudo-allergic reactions (due to cell lysis), lymphopenia, neutropenia and LON, hypogammaglobulinemia	Differential WBCC, serum immunoglobulins, CD19/20-positive B-cells count	Monitoring for infusion-related (allergic) reactions
Eculizumab	i. v., 900 mg 1x/week weeks 0–3, 1200 mg 1x/week week 4, thereafter 1200 mg every 2 weeks	Immediate within 1–2 weeks	Headaches, upper respiratory tract infections	Meningococcal infection and infections with other encapsulated bacteria	Anemia, leukopenia, fungal infections, infusion-related (allergic) reactions	BCC and differential WBCC	Patient teaching and close monitoring for meningococcal infection (exclusion before each infusion)
Ravulizumab	i. v., weight-based^2^, loading dose of 2400–3000 mg on day 1 followed by the maintenance dose (3000–3600 mg) after 2 weeks and then once every 8 weeks	Immediate within 1–2 weeks	Headaches, upper respiratory tract infections	Meningococcal infection and infections with other encapsulated bacteria	Anemia, leukopenia, fungal infections, infusion-related (allergic) reactions	BCC and differential WBCC	Patient teaching and close monitoring for meningococcal infection (exclusion before each infusion)
Tocilizumab	i. v., 6–8 mg/kg body weight, every 4–6 weeks*	After 12 to 24 weeks	Injection-related reactions, headache	Upper respiratory tract and urinary tract infections	Neutropenia, thrombocytopenia, elevation in liver enzymes, flare-up of chronic diverticulitis with potential gastrointestinal perforations, elevations in cholesterol or triglycerides, infusion-related (allergic) reactions	BCC and differential WBCC, liver enzymes, lipids	Clinical monitoring for infections due to suppression of CRP production in the context of infections
Satralizumab	s. c., 120 mg at weeks 0, 2, and 4, followed by every 4 weeks	After 12 to 24 weeks	Injection-related reactions, headache, arthralgia,	Mild to moderate infections, no opportunistic infections so far reported	Neutropenia, thrombocytopenia, elevation in liver enzymes, elevations in cholesterol or triglycerides, decrease in C3, C4 and fibrinogen	BCC and differential WBCC, liver enzymes, lipids	Clinical monitoring for infections due to suppression of CRP production in the context of infections

*BCC* blood cell count, *CRP* C-reactive protein, *IS* immunosuppressive therapies, *i. v.* intravenously, *LON* late-onset neutropenia, *PJP* pneumocystis jirovecii pneumonia, *PML* progressive multifocal leukoencephalopathy, *s. c.* subcutaneously, *WBCC* white blood cell count

^1^These aggregated recommendations do not include all potential side effects and do not replace the specific product information for each drug; ^2^for details see product information

*switch to s. c. application possible

**Table 2:** Main data of randomized, double-blind, placebo-controlled, time-to-event trials in NMOSD

Drug	Trial/Randomization	Number of patients / AQP4-IgG serostatus	Inclusion criteria		Concomitant immuno-suppression	Attacks n (%) (HR, 95% CI, and/or p)	previous immunotherapies	Duration of treatment in core study/open-label extension
			Previous disease activity	Age [years]				
Rituximab	RIN-1^1^/1:1	38 / all positive; including 11 AQP4-IgG negative patients who previously tested positive	Any history of optic neuritis or myelitis	16–80	No, but oral glucocorticoids, tapered during initiation period	0 vs. 7 (37%); (p = 0.0058)	0% with rituximab, other n.a	Median 72.1 weeks/mean 20.5 months (SD 10.1)^7^
Inebilizumab	N-MOmentum^2^/3:1	230 / 213-positive, 17-negative	≥ 1 attack in 12 months or ≥ 2 attacks in 24 months	> 18	No, but oral glucocorticoids during initiation period (20 mg/d until d14, then tapered to d21)	21 (12%) vs. 22 (39%);(0.272, 0.15–0.496, *p* < 0.0001)	Inebilizumab group: 66% (mostly azathioprine and glucocorticoids, including 7% with rituximab)	Up to 28 weeks/mean 3.2 years, up to 4.5 years (median)^8^
Eculizumab	PREVENT^3^/2:1	143 / all-positive	≥ 2 attacks in 12 months or ≥ 3 attacks in 24 months with 1 attack in the last 12 months	> 18	Yes	3 (3%) vs. 20 (43%); (0.06, 0.02–0.20, *p* < 0.001)	Eculizumab group: 78% IS at baseline; (27% with previous rituximab)	Median 89.4 weeks/median 132 weeks, up to 277 weeks^9^
Ravulizumab	CHAMPION–NMOSD^4^/Placebo group of PREVENT as external comparator	58 / all-positive	≥ 1 relapse in the last 12 months	> 18	Yes	0 vs. 29 (43%) in PREVENT (0.014, 0.000–0.103, *p* < 0.0001)	Ravulizumab group: 48% IS at baseline; 86% previuos IS (including 36% with previous rituximab)	Median 73.5 weeks (range 11.0–117.7)/OLE ongoing
Satralizumab	SAkuraSky^5^/1:1	83 /55 -positive, 28-negative	≥ 2 attacks in 24 months with 1 attack in the last 12 months	12–74	Yes	8 (20%) vs. 18 (43%); (0.38, 0.16–0.88, *p* = 0.02)	Satralizumab group: 78% with previous IS before add-on IS at baseline (including 4,9% with rituximab);	Median 107.4 weeks/median 4.4 years (range 0.1–7.0)^10^
	SAkuraStar^6^/2:1	95/63-positive, 31-negative	≥ 1 attack in 12 months	18–74	No	19 (30%) vs. 16 (50%); (0.45, 0.23–0.89, *p* = 0.018)	Satralizumab group: 87% with previous IS or other; 13% with previous B-cell depleting therapies	Median 92.3 weeks/median 4.0 years (range 0.1–6.1)^10^

*n.a.* not available, *HR* hazard ratio, *CI* confidence interval, *d* day, *OLE* open-label extension, *SD* standard deviation, *IS* immunosuppressive therapy

^1^Tahara 2020 The Lancet Neurology [216]

^2^Cree 2019 The Lancet [51]

^3^Pittock 2019 N Eng J Med [179]

^4^Pittock 2023 Ann Neurol [178]

^5^Yamamura 2019 N Eng J Med [250]

^6^Traboulsee 2020 The Lancet Neurol [223]

^7^Tahara 2022 MSRD [217]

^8^Rensel 2022 MSJ [188]

^9^ Wingerchuk 2021 Ann Neurol [246]

^10^Yamamura 2022 MSRD [251]

