# Supplementary material for: Update on the diagnosis and treatment of neuromyelitis optica spectrum disorders (NMOSD) – revised recommendations of the Neuromyelitis Optica Study Group (NEMOS). Part II: Attack therapy and long-term management
Source: J Neurol. 2023 Sep 7;271(1):141–76. doi: 10.1007/s00415-023-11910-z (PMC10770020; doi:10.1007/s00415-023-11910-z)
Supplement: Supplementary file 1 — Supplementary file1 (DOCX 21 KB) [file 415_2023_11910_MOESM1_ESM.docx]

**SUPPLEMENT: Survey results**

**Title:**

**Update on the diagnosis and treatment of neuromyelitis optica spectrum disorders (NMOSD) - revised recommendations of the Neuromyelitis Optica Study Group (NEMOS).**

**Part II: Attack therapy and long-term management**

**Authors:**

Tania Kümpfel^1^, Katrin Giglhuber^2^, Orhan Aktas^3^, Ilya Ayzenberg^4^, Judith Bellmann-Strobl^5,6,7,8^, Vivien Häußler^9^, Joachim Havla^1^, Kerstin Hellwig^4^, Martin W. Hümmert^10^, Sven Jarius^11^, Ingo Kleiter^4,12^, Luisa Klotz^13^, Markus Krumbholz^14,15,16^, Friedemann Paul^5,6,7,8^, Marius Ringelstein^3,17^, Klemens Ruprecht^5^, Makbule Senel^18^, Jan-Patrick Stellmann^9,19,20^, Florian Then Bergh^21^, Corinna Trebst^11^, Hayrettin Tumani^19^, Clemens Warnke^22^, Brigitte Wildemann^12^, Achim Berthele^2^; on behalf of the Neuromyelitis Optica Study Group (NEMOS)

^1^ Institute of Clinical Neuroimmunology, LMU Hospital, Ludwig-Maximilians-Universität

München, Munich, Germany

^2^ Department of Neurology, School of Medicine, Technical University Munich, Klinikum rechts

der Isar, Munich, Germany

^3^ Department of Neurology, Medical Faculty, Heinrich Heine University Düsseldorf, Düsseldorf,

Germany

^4^ Department of Neurology, St. Josef Hospital, Ruhr University Bochum, Bochum, Germany

^5^ Department of Neurology, Charité – Universitätsmedizin Berlin, corporate member of Freie

Universität Berlin and Humboldt-Universität zu Berlin, Berlin, Germany

^6^ Experimental and Clinical Research Center, a cooperation between the Max Delbrück Center

for Molecular Medicine in the Helmholtz Association and Charité – Universitätsmedizin Berlin,

Germany

^7^ Max Delbrück Center for Molecular Medicine in the Helmholtz Association (MDC), Berlin,

Germany

^8^ NeuroCure Clinical Research Center, Charité Universitätsmedizin Berlin, corporate member

of Freie Universität Berlin and Humboldt-Universität zu Berlin, and Berlin Institute of Health,

and Max Delbrück Center for Molecular Medicine, Berlin, Germany

^9^ Department of Neurology and Institute of Neuroimmunology and MS (INIMS), University

Medical Center Hamburg-Eppendorf, Hamburg, Germany

^10^ Department of Neurology, Hannover Medical School, Hannover, Germany

^11^ Molecular Neuroimmunology Group, Department of Neurology, University of Heidelberg,

Heidelberg, Germany

^12^ Marianne-Strauß-Klinik, Behandlungszentrum Kempfenhausen für Multiple Sklerose Kranke,

Berg, Germany

^13^ Department of Neurology with Institute of Translational Neurology, University of Münster,

Münster, Germany

^14^ Department of Neurology and Pain Treatment, Immanuel Klinik Rüdersdorf, University

Hospital of the Brandenburg Medical School Theodor Fontane, Rüdersdorf bei Berlin, Germany

^15^ Faculty of Health Sciences Brandenburg, Brandenburg Medical School Theodor Fontane,

Rüdersdorf bei Berlin, Germany

^16^ Department of Neurology & Stroke, University Hospital of Tübingen, Tübingen, Germany

^17^ Department of Neurology, Center for Neurology and Neuropsychiatry, LVR-Klinikum, Heinrich

Heine University Düsseldorf, Düsseldorf, Germany

^18^ Department of Neurology, University of Ulm, Ulm, Germany

^19^ APHM, Hopital de la Timone, CEMEREM, Marseille, France

^20^ Aix Marseille Univ, CNRS, CRMBM, Marseille, France

^21^ Department of Neurology, University of Leipzig, Leipzig, Germany

^22^ Department of Neurology, Faculty of Medicine and University Hospital Cologne, University of Cologne, Cologne, Germany

* Corresponding authors:

Tania Kümpfel: Tania.Kuempfel@med.uni-muenchen.de

Achim Berthele: achim.berthele@tum.de

**Survey results**

Results of the DELPHI survey for the recommendations A1-B29. An approval of >75% was rated as consent.

| Recommendation | Number of votes | Number of approvals | Number of rejections | Approval [%] |
| --- | --- | --- | --- | --- |
| A1 | 24 | 24 | 0 | 100 |
| A2 | 24 | 22 | 2 | 92 |
| A3 | 24 | 24 | 0 | 100 |
| A4 | 24 | 22 | 2 | 92 |
| A5 | 24 | 22 | 2 | 92 |
| A6 | 24 | 23 | 1 | 96 |
| B1 | 24 | 24 | 0 | 100 |
| B2 | 24 | 21 | 3 | 88 |
| B3 | 24 | 22 | 2 | 92 |
| B4 | 24 | 24 | 0 | 100 |
| B5 | 24 | 22 | 2 | 92 |
| B6 | 24 | 23 | 1 | 96 |
| B7 | 24 | 24 | 0 | 100 |
| B8 | 24 | 23 | 1 | 96 |
| B9 | 24 | 24 | 0 | 100 |
| B10 | 24 | 22 | 2 | 92 |
| B11 | 24 | 24 | 0 | 100 |
| B12 | 24 | 23 | 1 | 96 |
| B13 | 24 | 23 | 1 | 96 |
| B14 | 24 | 23 | 1 | 96 |
| B15 | 24 | 22 | 2 | 92 |
| B16 | 24 | 21 | 3 | 88 |
| B17 | 24 | 22 | 2 | 92 |
| B18 | 24 | 23 | 1 | 96 |
| B19 | 24 | 24 | 0 | 100 |
| B20 | 24 | 24 | 0 | 100 |
| B21 | 24 | 19 | 5 | 79 |
| B22 | 24 | 24 | 0 | 100 |
| B23 | 24 | 23 | 1 | 96 |
| B24 | 24 | 23 | 1 | 96 |
| B25 | 24 | 20 | 4 | 83 |
| B26 | 24 | 24 | 0 | 100 |
| B27 | 24 | 22 | 2 | 92 |
| B28 | 24 | 23 | 1 | 96 |
| B29 | 24 | 23 | 1 | 96 |

The implementation of ravulizumab in the recommendations was voted additionally and separately in April 2023 after the CHMP of the EMA recommended approval for ravulizumab for treatment of AQP-4-IgG positive NMOSD in April 2023. 23/24 authors consented (96%) to include ravulizumab in the manuscript and recommendations.
